# Patient-Centered Fertility Care in Kazakhstan: Preliminary Cultural Adaptation of a Fertility-Care Survey and Patient–Staff Perception Gaps in Two Assisted-Reproduction Clinics

**DOI:** 10.3390/healthcare14182941

**Published:** 2026-09-10

**Authors:** Aigerim T. Kushtekova, Maral G. Nogayeva, Lyudmila S. Yermukhanova, Vyacheslav N. Lokshin, Ardak N. Nurbakyt, Aigul Y. Tazhiyeva, Aiman A. Musina, Akmaral K. Mussakhanova, Alireza Afshar

**Affiliations:** 1 Department of Public Health and Public Health Care, West-Kazakhstan Marat Ospanov Medical University, Aktobe 30012, Kazakhstan; aikerim0105@gmail.com; 2 Department of Rheumatology, Asfendiyarov Kazakh National Medical University, Almaty 050012, Kazakhstan; maralnogaeva654@gmail.com; 3 International Center for Clinical Reproduction «Persona», National Academy of Sciences of the Republic of Kazakhstan, Astana 010000, Kazakhstan; v.lokshin@persona-ivf.kz; 4 Department of Public Health, Asfendiyarov Kazakh National Medical University, Almaty 050012, Kazakhstan; a.nurbakyt@kaznmu.kz; 5 Department of General Medical, Asfendiyarov Kazakh National Medical University, Almaty 050012, Kazakhstan; aigul.esentaikyzy@gmail.com; 6 Department of Epidemiology and Biostatistics, Non-Profit Joint-Stock Company in Astana Medical University, Astana 010000, Kazakhstan; mussina.a@amu.kz; 7 Department of Public Health and Management, Non-Profit Joint-Stock Company in Astana Medical University, Astana 010000, Kazakhstan; makmaral1@gmail.com; 8 Student Research Committee, Bushehr University of Medical Sciences, Bushehr 7514633341, Iran

**Keywords:** patient-centered care, infertility, assisted reproductive technology, fertility care, patient experience, healthcare staff, patient–staff perception gap

## Abstract

**Highlights:**

**What are the main findings?**

- Patient and staff perceptions of fertility care differed substantially in both directions, with the largest gaps involving cost, communication, information, participation, privacy, emotional support, and continuity of care.
- Patient-defined paired priority gaps highlighted privacy, cost, information provision, and participation in care as important areas for improvement, while several survey domains showed limited internal consistency and global satisfaction ratings demonstrated a strong ceiling effect.

**What are the implications of the main findings?**

- Fertility clinics should not rely on staff assessments alone when evaluating patient-centered care and should integrate patient-reported experience and importance ratings into routine quality-improvement activities.
- The exploratory survey can help identify preliminary service-level priorities, but further psychometric refinement and broader multicenter validation are needed before its domain scores are interpreted as established measurement scales.

**Abstract:**

**Background:** Patient-centered care is particularly important in assisted reproductive technology, where treatment is clinically demanding and emotionally intensive. This study documented a preliminary cultural and linguistic adaptation of a fertility-care survey for Kazakhstan and evaluated patient–staff perception gaps, item-level priorities, preliminary reliability, and exploratory associations with global care ratings. **Methods:** A cross-sectional survey was conducted in two assisted-reproduction clinics in Almaty. The analytic workbook contained 273 patient and 74 staff records; 173 patient response patterns were unique and 100 records matched an earlier exact pattern. Group-level patient–staff comparisons between independent respondent samples used prespecified harmonized indices, Welch tests, false-discovery-rate correction, clinic-stratified analyses, and one-pattern-per-record sensitivity analyses. Importance–experience differences were calculated within respondents with both values available. Global-rating regressions used HC3 robust inference and were treated as exploratory. **Results:** Staff rated cost, attitude and communication, information and education, and participation more favorably, whereas patients rated privacy, emotional support, and continuity more favorably. Accessibility and competence showed little difference. Clinic-stratified and one-pattern-per-record analyses preserved the principal contrast directions, although the Physical Comfort proxy was not robust. Several indices showed weak or negative internal consistency. Global ratings had a marked ceiling, with 79.1% at 10/10, and the HC3 joint test for the added care-index block was not significant (*p* = 0.080). **Conclusions:** This exploratory survey identified substantial, bidirectional group-level patient–staff differences and concrete item-level priorities, but the scoring and psychometric evidence are insufficient to interpret the domain indices as established measurement scales. Source-codebook verification and broader structural validation are required.

## 1. Introduction

Patient-centered care (PCC) has been widely recognized as a core dimension of healthcare quality, defined by the Institute of Medicine as care that “is respectful of and responsive to individual patient preferences, needs, and values, and ensures that patient values guide all clinical decisions” [1,2]. In fertility medicine, where treatment regimens such as in vitro fertilization (IVF) and intracytoplasmic sperm injection (ICSI) involve complex protocols, substantial financial investment, and considerable emotional strain, the principles of PCC are particularly critical to optimize clinical outcomes, alleviate psychological distress, and reduce treatment discontinuation [3,4]. Infertility, affecting up to 15% of couples worldwide, imposes profound psychosocial and economic burdens, including anxiety, depression, relationship stress, and catastrophic healthcare expenditures [5,6].

Assisted reproductive technologies (ART) have transformed the landscape of infertility treatment, offering hope to many couples but introducing additional challenges: variable success rates, lengthy treatment cycles, and high out-of-pocket costs [7]. Research demonstrates that clear communication, respect for patient autonomy, and timely emotional support are associated with better adherence to treatment protocols, enhanced psychological well-being, and improved perceived quality of care [8,9]. Conversely, inadequate information provision and lack of empathy correlate with increased cancelation rates, heightened anxiety, and lower overall satisfaction [10].

To systematically assess PCC in infertility settings, several patient-reported instruments have been developed. The Patient-Centeredness Questionnaire for Infertility was developed in the Netherlands from patient focus groups and subsequently validated as a 46-item instrument with seven subscales, using paired experience and importance ratings for relevant care aspects [11]. Related qualitative and measurement work in other settings has reinforced that the meaning and priority of patient-centered fertility-care domains can vary across healthcare systems and cultural contexts [12,13,14].

Despite global advances, Central Asia remains under-represented in PCC research. Kazakhstan, with an estimated infertility prevalence of 12–15.5% among couples, introduced modern ART in the mid-1990s and has expanded to over 20 clinics nationwide. Yet per capita utilization remains limited, and national ART registries are incomplete [15,16]. Cultural imperatives around childbearing in Kazakh society amplify the psychosocial stakes of infertility; societal stigma and marital strain are common for childless couples [17]. Quality-of-life research using the FertiQoL instrument revealed that Kazakhstani IVF patients report lower quality-of-life scores compared to European counterparts, particularly among lower-income groups, suggesting that financial and systemic barriers exacerbate distress [18].

To date, no culturally adapted patient-centered fertility-care instrument has been reported for routine use in Kazakhstan. Webair et al. [1] developed the Patient-Centered Infertility Questionnaire for Female Clients (PCIQ-F) in an Arabic context. The draft instrument retained 57 patient-centered care experience items across ten domains, together with background variables and a global care-quality item. Its domain framework provides a useful basis for cross-cultural work, but local adaptation, transparent scoring, and additional psychometric evaluation are required before broad implementation in Kazakhstan.

This study aimed to document the preliminary cultural and linguistic adaptation of a patient-centered fertility-care survey for use in Kazakhstan, describe preliminary internal consistency and item behavior, compare patient experiences with staff perceptions across prespecified matched care-domain indices, identify patient-reported quality-improvement priorities, and explore, as secondary analyses, associations between patient-centered care domains and patients’ global ratings of fertility care.

## 2. Materials and Methods

### 2.1. Study Design and Setting

This study was a cross-sectional analytical survey conducted as part of a preliminary cultural adaptation project on patient-centered fertility care in Almaty, Kazakhstan. Data were collected between March and May 2025 in two assisted-reproduction clinics, the International Clinical Center for Reproductology Persona and the Institute of Reproductive Medicine. Patient and staff questionnaires were analyzed as independent samples because individual patient responses were not paired with specific staff respondents. Accordingly, all patient–staff differences represent group-level contrasts between independent respondent samples, not provider–patient dyadic discrepancies. The reporting structure was informed by the STROBE recommendations for cross-sectional observational studies [19]. The reporting revision also considered the Consensus-Based Checklist for Reporting of Survey Studies (CROSS) [20].

### 2.2. Survey Framework and Cultural Adaptation

The Kazakhstan survey was anchored in the ten-domain patient-centered infertility-care framework represented in the PCIQ-F, including Accessibility, Minimizing Cost, Physical Comfort, Privacy, Staff Attitude and Communication, Staff Competence, Information and Education, Emotional and Psychological Support, Continuity and Coordination, and Participation in Care. The source PCIQ-F development study retained 57 care-experience items after face and content review, in addition to background questions and a global care-quality item [1].

The administered survey also retained the paired experience and importance logic used in the PCQ-Infertility tradition, in which patients evaluate both what they experienced and, for applicable items, how important that aspect of care was to them. This structure was preserved because it supports both measurement of reported care and identification of patient-defined quality-improvement priorities [11].

Available study documentation recorded forward translation of the item pool into Kazakh and Russian, reconciliation of wording, back translation, cognitive debriefing with five fertility patients, and review by a ten-member multidisciplinary panel. The preserved records did not identify the translators’ professional qualifications, independence, source-to-target assignments, the item-level reconciliation decisions, or the specific wording changes arising from the cognitive interviews; these details therefore could not be reconstructed. These steps are consistent with established principles for cross-cultural adaptation of self-report instruments, which emphasize translation, synthesis, back translation, expert review, and pretesting in the target population [21,22].

The reproducible analysis focused on survey responses, matched patient–staff comparisons, paired patient importance–experience differences, and preliminary reliability and item behavior. No Kazakhstan-specific quantitative expert-rating dataset was available, and structural validity, test–retest reliability, measurement invariance, and responsiveness were not evaluated; consequently, item-relevance statistics and claims of psychometric validation were not reported. The distinction between cultural adaptation and complete validation is consistent with contemporary measurement-property guidance [18]. Scale-development guidance likewise treats pretesting, factor extraction, reliability, and validity as distinct stages [23].

### 2.3. Participants and Data Collection

Patients were eligible if they were women aged 18 years or older, were receiving or preparing to receive fertility treatment at one of the participating clinics, and were able to complete the questionnaire in Kazakh or Russian. The staff sample included physicians, nurses, embryology and laboratory personnel, and other staff directly involved in fertility services. Participants completed the survey after informed consent.

The archived study materials available for this revision did not record how eligible participants were approached, whether recruitment was consecutive, the number approached or declining participation, or a response-rate denominator. The sampling approach therefore cannot be classified more specifically than clinic-based recruitment, and a response rate cannot be calculated. No a priori sample-size calculation was documented; this exploratory analysis used all analyzable questionnaires collected during the study period, and confidence intervals are reported to show estimate precision [20].

The reproducible analytic workbook contained 273 patient records and 74 staff records. Patient variables included clinic, education, current or recent treatment, age category, ethnicity, pregnancy history, parental history, infertility cause, care-experience items, paired importance items where available, and a global rating of fertility care with 10 representing the highest rating. Staff variables included clinic, education, profession, age category, gender, work experience, ethnicity, and corresponding care-domain items. The characteristics included in the final analysis are summarized in Table 1.

### 2.4. Item Scoring and Construction of Domain Scores

The administered patient and staff questionnaires used heterogeneous, item-specific response formats rather than a single uniform Likert scale, and corresponding patient and staff items did not always have identical category structures. To make the prespecified domain indices directionally interpretable across questions, care-experience responses were harmonized to a 0–3 metric, with higher values consistently indicating a more favorable care experience. Positively worded four-category items were mapped from 0 to 3, negatively worded items were reverse scored, binary items were mapped to the endpoints of the same scale, and three-category ordered responses were linearly mapped to 0, 1.5, and 3. Item-specific response categories indicating that the question did not apply, that the respondent did not know, or that the experience had not occurred were treated as missing rather than as poor care. Semantic item matching and harmonization enabled directional group-level comparisons, but they did not establish psychometric equivalence between the patient and staff questionnaires.

Prespecified domain indices were calculated as the mean of eligible scored items when more than 50% of the items assigned to that domain were available for a respondent. Semantic equivalence was operationalized item by item: patient and staff questions were matched only when their English labels referred to the same service feature and could be oriented in the same favorable-care direction. This content mapping was encoded in the reproducible script and is reported with item-level results in Supplementary Table S9; it was not a formal test of measurement invariance. The available Physical Comfort measure consisted of a waiting-room item rather than a complete multi-item comfort scale and was therefore labeled Physical Comfort proxy throughout the analysis. Single-item or proxy constructs were not assigned internal-consistency coefficients.

Patient importance responses were transformed to the same 0–3 orientation, with higher scores representing greater importance. For each item, an exploratory priority difference was calculated within patients who had both an experience and an importance value. The paired analysis reported the complete-pair sample size, paired experience and importance means, mean within-person difference, 95% confidence interval, paired t test, and false-discovery-rate-adjusted *p* value. Positive differences indicated aspects considered more important than favorably experienced. This importance–experience approach is conceptually aligned with the original PCQ-Infertility measurement strategy [11].

### 2.5. Data-Quality Assessment and Sensitivity Datasets

Before inferential analysis, the data were screened for missingness, out-of-range values, zero-variance variables, and exact response patterns. Values outside the permitted range for a given item were set to missing only according to prespecified item-level rules. Staff global care ratings were not analyzed because 50 of 74 records contained code 11 despite the stated 0–10 range; without a linkable source form or codebook confirming its meaning, code 11 was treated as invalid for this variable.

The preserved project records did not document whether questionnaires were completed on paper or electronically, how responses were transferred into the analytical workbook, or whether double entry or record-level source verification was used. The workbook contained no participant identifiers or timestamps that could link exact response patterns or staff code 11 to source forms. An expanded scoring audit identified 308 item responses outside the implemented scoring sets, comprising 278 patient and 30 staff responses. The patient total included 240 importance responses, of which 206 occurred in P39; because the corresponding experience item P38 was not scored, P39 was not included in the paired priority analysis. Across patient and staff experience items alone, 68 unexpected responses were identified, comprising 38 patient and 30 staff responses. The meaning of every unexpected value was undocumented in the available codebook and archived materials, so each was set to missing for the affected item. The audit also confirmed that staff global-rating code 11 was undocumented and was treated as missing, with the staff global rating excluded from analysis. These treatments were already implemented in the reported analyses and did not alter the numerical results. Because the administered item-level codebook was unavailable, all harmonized-score analyses are conditional on the explicit mapping in Supplementary Code S1 (Supplementary Tables S3 and S12).

Exact-response-pattern screening identified 173 unique response patterns among the 273 available patient records. Some records therefore contained identical values across all variables included in the analytic dataset. Because the reproducible workbook did not contain unique respondent identifiers, submission timestamps, or linkable source-level identifiers, record-level verification against original questionnaires could not be performed and it was not possible to determine whether identical patterns represented different participants or repeated records. In the absence of evidence establishing that these records were erroneous duplicates, all 273 available patient records were retained in the primary analyses. To evaluate whether the findings were sensitive to repeated exact-response patterns, the principal patient–staff comparisons and the multivariable global-rating model were repeated after retaining the first occurrence of each exact response pattern in the original workbook row order. This reduced dataset was used exclusively as a sensitivity analysis and was not considered evidence that the excluded records represented duplicate participants.

### 2.6. Statistical Analysis

All analyses were performed in R version 4.3.3 (R Foundation for Statistical Computing, Vienna, Austria) using reproducible scripts and packages for data management, psychometric summaries, robust inference, and graphical presentation (Supplementary Table S1 and Supplementary Code S1). R is an open-source statistical computing environment widely used for reproducible quantitative research [24].

Categorical variables were summarized as frequencies and percentages. Continuous and harmonized domain scores were described using the mean, standard deviation, median, interquartile range, minimum, maximum, and floor and ceiling percentages when appropriate. Internal consistency was evaluated for eligible multi-item indices using Cronbach alpha, standardized alpha, and McDonald omega; corrected item-total correlations, alpha if deleted, and pairwise inter-item correlations were also examined. These statistics were treated as item and index diagnostics rather than proof of unidimensionality or validity, and low-reliability indices were retained only as prespecified descriptive summaries alongside item-level results [25,26].

Patient and staff prespecified domain indices were compared with Welch independent-samples t tests because the groups were independent and differed in size and variance. For each index, the analysis reported the patient and staff means, the mean difference calculated as staff minus patient, a 95% confidence interval, and Hedges g as a small-sample-corrected standardized mean difference [27,28].

Because respondents were drawn from two clinics, the patient–staff comparisons were repeated within each clinic and again within each clinic after retaining one patient record per unique exact response pattern. With only two clinics, a random-effects multilevel model was not fitted because clinic-level variance could not be estimated reliably; the clinic-stratified results were treated as sensitivity analyses rather than independent estimates of clinic effects (Supplementary Table S8).

Because multiple domain comparisons were performed, the Benjamini–Hochberg procedure was applied to the Welch-test *p* values to control the false discovery rate. Statistical evidence for patient–staff differences was evaluated using the adjusted *p* values together with effect sizes and confidence intervals rather than by *p* values alone [29].

Associations with the patient global care rating were explored using hierarchical ordinary least-squares regression. Model 1 included age category, education, and clinic affiliation. Model 2 added all prespecified patient-centered care indices. Both models were fitted to the same complete-case sample to make the change in explained variance directly comparable. Unstandardized coefficients were reported with HC3 heteroskedasticity-robust standard errors and 95% confidence intervals. HC3 was used to reduce sensitivity of coefficient inference to heteroskedastic residual variance, and the implementation followed heteroskedasticity-consistent covariance estimation available in R [30,31].

Incremental model contribution was summarized using R-squared, adjusted R-squared, change in R-squared, the conventional nested-model F-change statistic, and an HC3 robust Wald test for the block of added patient-centered care domains. Because the global rating showed a pronounced ceiling distribution, a secondary top-box logistic regression contrasted a rating of 10 with ratings below 10. The top-box model used the same predictor structure and HC3 robust coefficient inference. It contained 207 top-box and 46 below-top-box observations with 14 non-intercept predictor parameters, or 3.3 observations in the smaller outcome group per predictor parameter. One-pattern-per-record sensitivity analyses were used to evaluate the stability of the main patient–staff contrasts and the multivariable satisfaction findings. All tests were two-sided, with a nominal alpha level of 0.05; false-discovery-rate adjustment was applied to the primary family of patient–staff index comparisons. All global-rating models were explicitly exploratory because of the extreme ceiling and limited information in the lower-frequency outcome category.

### 2.7. Ethical Considerations

The study received institutional ethics approval by Local Bioethics Commission of NJSC “West Kazakhstan Marat Ospanov Medical University” under Protocol No. 2 (assigned No. 2.2). Participation was voluntary and informed consent was obtained before questionnaire completion. The statistical analysis was performed on de-identified survey records.

## 3. Results

### 3.1. The Analytic Sample Reflected Predominantly Active ART Treatment and a Broad Staff Mix

The reproducible dataset included 273 patient records and 74 staff records. Among the 272 patients with recorded clinic affiliation, 145 (53.3%) attended Persona and 127 (46.7%) attended IRM. Most patients had higher professional or university education (57.1%), while 27.5% had secondary or vocational secondary education, 13.9% had postgraduate education, and 1.5% were coded as other. IVF or ICSI was the current or most recent treatment for 228 patients (83.5%), 22 (8.1%) had not yet started treatment, and 23 (8.4%) reported another ART or treatment pathway. More than half of the patients were in the 30–34 year category (52.0%), 64.5% were recorded as Kazakh, and female-factor infertility was the most frequently recorded cause (52.0%), followed by unexplained infertility (18.7%), male-factor infertility (17.9%), both male and female factors (8.4%), and unknown cause (2.9%) (Table 1).

The staff sample included 43 participants from Persona (58.1%) and 31 from IRM (41.9%). Doctors accounted for 40.5% of staff, nurses for 37.8%, embryologists and laboratory technicians for 9.5% each, and other staff for 2.7%. Just over half had higher professional or university education (56.8%), 83.8% were women, and work experience was distributed across all categories from less than one year to more than 20 years (Table 1).

The patient domain distributions were heterogeneous. Staff Competence had the highest patient mean (2.401, SD 0.587), followed by Continuity and Coordination (2.285, SD 0.584), whereas Staff Attitude and Communication (1.020, SD 0.486) and Minimizing Cost (1.049, SD 0.897) had the lowest patient means. In the staff sample, Staff Competence (2.385, SD 0.457), Information and Education (2.310, SD 0.500), and Minimizing Cost (2.291, SD 0.832) were among the highest-rated domains, whereas Continuity and Coordination had the lowest staff mean (1.457, SD 0.427). Floor and ceiling patterns were also uneven, including a 37.4% patient floor for Minimizing Cost, a 32.6% patient ceiling for Staff Competence, a 55.4% staff ceiling for Minimizing Cost, and a 45.3% staff ceiling for the Physical Comfort proxy (Supplementary Table S2).

Exact-response-pattern screening identified 173 unique patterns among the 273 patient records. All 145 Persona records had unique exact patterns. The 127 IRM records comprised 27 unique exact patterns and therefore accounted for all 100 records beyond the first occurrence of each pattern; the single record without a clinic designation was unique. Because respondent-level identifiers were unavailable, these matches could not be classified as either independent participants with identical responses or duplicate records. Accordingly, all 273 records were retained for the primary analyses, and a reduced dataset containing the first occurrence of each exact response pattern in the original workbook row order was analyzed separately to assess sensitivity to these matches (Supplementary Tables S3, S5, S6 and S8). The patient global rating had a mean of 9.59 (SD 1.14), a median of 10, and an interquartile range of 10–10; 79.1% of patients selected the maximum rating of 10. Fifty of 74 staff global ratings were outside the intended scale because they were coded as 11, so staff global satisfaction was excluded from the analysis (Supplementary Table S3).

### 3.2. Group-Level Patient–Staff Differences Were Large but Clearly Bidirectional Across Care Domains

Matched-domain comparisons showed that staff ratings were substantially more favorable than patient ratings for several service dimensions. The largest positive staff–patient difference was observed for Minimizing Cost, with patient and staff means of 1.049 and 2.291, respectively, giving a mean difference of 1.241 (95% CI 1.022 to 1.461; Hedges g = 1.401; FDR-adjusted *p* < 0.001). Staff Attitude and Communication showed an even larger standardized separation, with a mean difference of 1.130 (95% CI 1.047 to 1.213; g = 2.520; FDR-adjusted *p* < 0.001). Information and Education also favored staff ratings by 0.981 points (95% CI 0.851 to 1.112; g = 1.914; FDR-adjusted *p* < 0.001), while Participation in Care favored staff by 0.579 points (95% CI 0.447 to 0.710; g = 1.268; FDR-adjusted *p* < 0.001) (Table 2).

The direction of discordance was reversed for three domains. Patients rated Continuity and Coordination more favorably than staff did, producing a staff-minus-patient difference of −0.829 (95% CI −0.949 to −0.708; g = −1.492; FDR-adjusted *p* < 0.001). Patient ratings were also higher for Privacy (difference −0.399, 95% CI −0.539 to −0.258; g = −0.662; FDR-adjusted *p* < 0.001) and Emotional and Psychological Support (difference −0.228, 95% CI −0.350 to −0.107; g = −0.455; FDR-adjusted *p* < 0.001).

**Figure 1:**
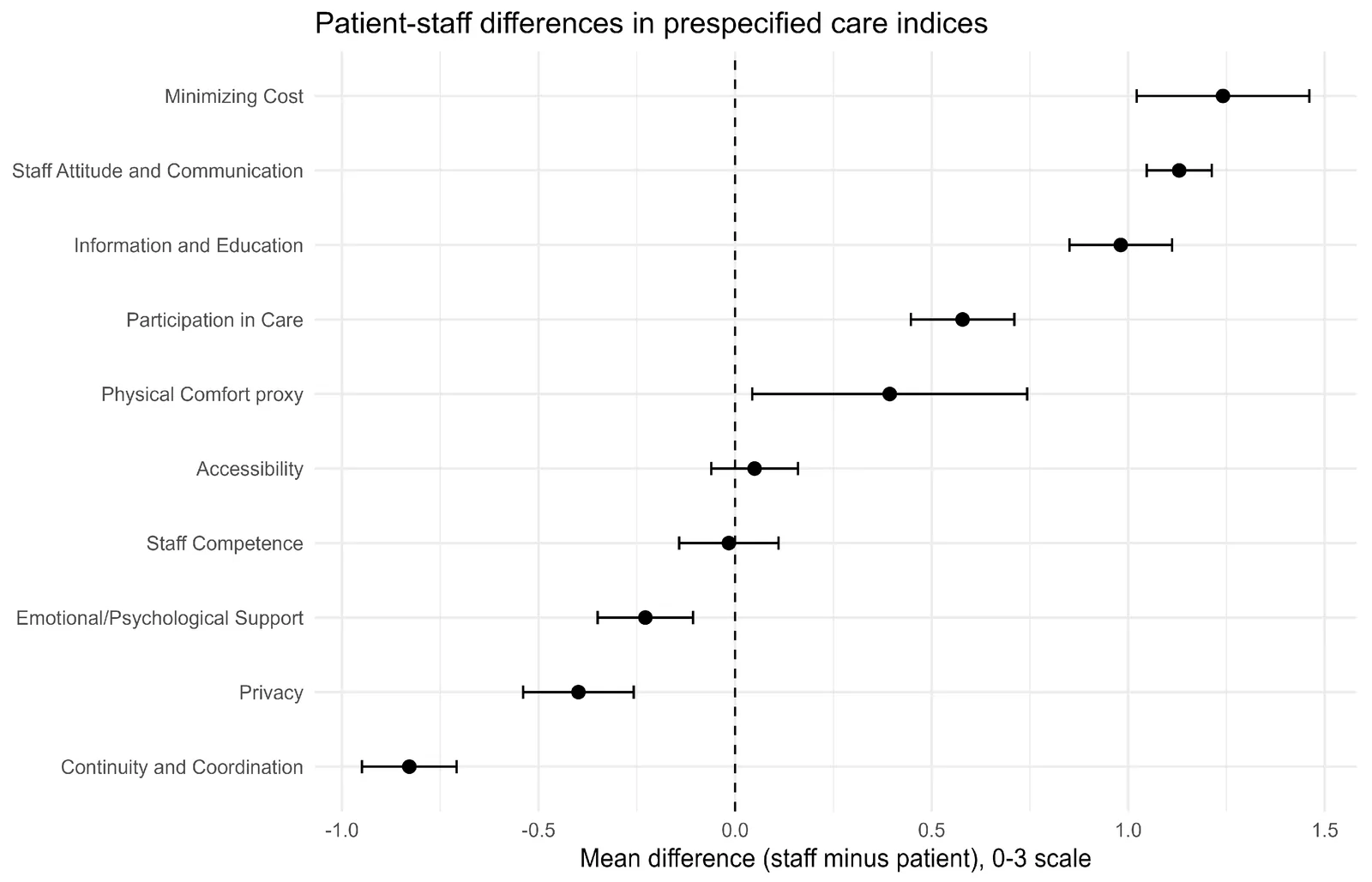
Patient–staff differences in prespecified matched patient-centered fertility-care indices. Mean differences are calculated as staff minus patient scores on the harmonized 0–3 scale. Positive values indicate more favorable staff ratings, whereas negative values indicate more favorable patient ratings. Horizontal bars represent 95% confidence intervals from Welch independent-samples comparisons. The vertical dashed line at zero represents no patient–staff difference.

Accessibility showed little evidence of a patient–staff difference (2.147 versus 2.197; difference 0.050, 95% CI −0.061 to 0.160; g = 0.101; FDR-adjusted *p* = 0.418), and Staff Competence was nearly identical between groups (2.401 versus 2.385; difference −0.016, 95% CI −0.142 to 0.110; g = −0.028; FDR-adjusted *p* = 0.803). The Physical Comfort proxy showed a smaller staff-favorable difference of 0.393 points (95% CI 0.044 to 0.743; g = 0.342; FDR-adjusted *p* = 0.035), although this contrast did not remain statistically significant in the one-pattern-per-record sensitivity analysis. The magnitude and direction of all primary domain contrasts are shown visually in Figure 1.

Clinic-stratified analyses showed that the principal differences were not explained solely by the unequal clinic composition. In Persona, staff ratings were higher for cost, Physical Comfort proxy, attitude and communication, information and education, and participation, while patient ratings were higher for privacy, emotional support, and continuity. In IRM, the same major differences were observed except that Physical Comfort proxy (difference −0.018; FDR *p* = 0.944) and emotional support (difference −0.176; FDR *p* = 0.114) showed no evidence of a patient–staff difference. The IRM one-pattern-per-record analysis reproduced this pattern, with Physical Comfort proxy and emotional support remaining nonsignificant (Supplementary Table S8).

### 3.3. Internal Consistency Was Heterogeneous and Weak for Several Domains

Internal-consistency estimates varied considerably between domains and respondent groups. Among patients, the strongest omega estimates were observed for Information and Education (omega = 0.787) and Staff Attitude and Communication (omega = 0.784), although their raw Cronbach alpha values were only 0.591 and 0.605. Continuity and Coordination showed alpha = 0.647 and omega = 0.674. Emotional and Psychological Support had alpha = 0.441 and omega = 0.647, while Staff Competence had alpha = 0.393 and omega = 0.495 (Table 3).

Several prespecified indices showed poor internal consistency. Patient Accessibility had alpha = 0.252 and omega = 0.307, Privacy had alpha = 0.204 and omega = 0.564, and Participation in Care produced a negative alpha of −0.238 with omega = 0.277. In the staff sample, Information and Education showed the highest alpha (0.615) and omega = 0.652, followed by Emotional and Psychological Support with alpha = 0.547 and omega = 0.633. Other staff-domain alpha values were low, including Accessibility (0.157), Privacy (0.175), Staff Competence (0.299), and Participation in Care (0.304). Staff Continuity and Coordination showed a marked discrepancy between alpha = 0.213 and omega = 0.729. For patient Participation in Care, the three pairwise item correlations were −0.180, −0.044, and 0.020, and all corrected item-total correlations were negative. No items were deleted post hoc to improve alpha. Item diagnostics and inter-item correlations are reported in Supplementary Tables S10 and S11, and item-level patient–staff comparisons are reported in Supplementary Table S9. Accordingly, the domain means and between-group differences are presented as exploratory comparisons of prespecified descriptive composites, not as estimates from established reflective scales.

### 3.4. Importance–Experience Gaps Identified Concrete Patient-Defined Improvement Priorities

The paired importance–experience analysis identified several items whose importance ratings exceeded the favorability of the corresponding experience. The largest mean within-person difference was for Privacy Q115 (*n* = 215; experience 0.595; importance 2.274; difference 1.679; 95% CI 1.479 to 1.879). The next largest differences were Minimizing Cost Q107 (*n* = 271; 1.343; 95% CI 1.195 to 1.491), Information and Education Q36 (*n* = 272; 1.331; 95% CI 1.174 to 1.488), Participation in Care Q55 (*n* = 272; 1.180; 95% CI 1.068 to 1.293), and Information and Education Q30 (*n* = 241; 0.925; 95% CI 0.742 to 1.109). Other leading positive differences were Q32 (0.690), Q63 (0.648), Q103 (0.576), Q99 (0.505), and Q26 (0.458) (Supplementary Table S4).

**Figure 2:**
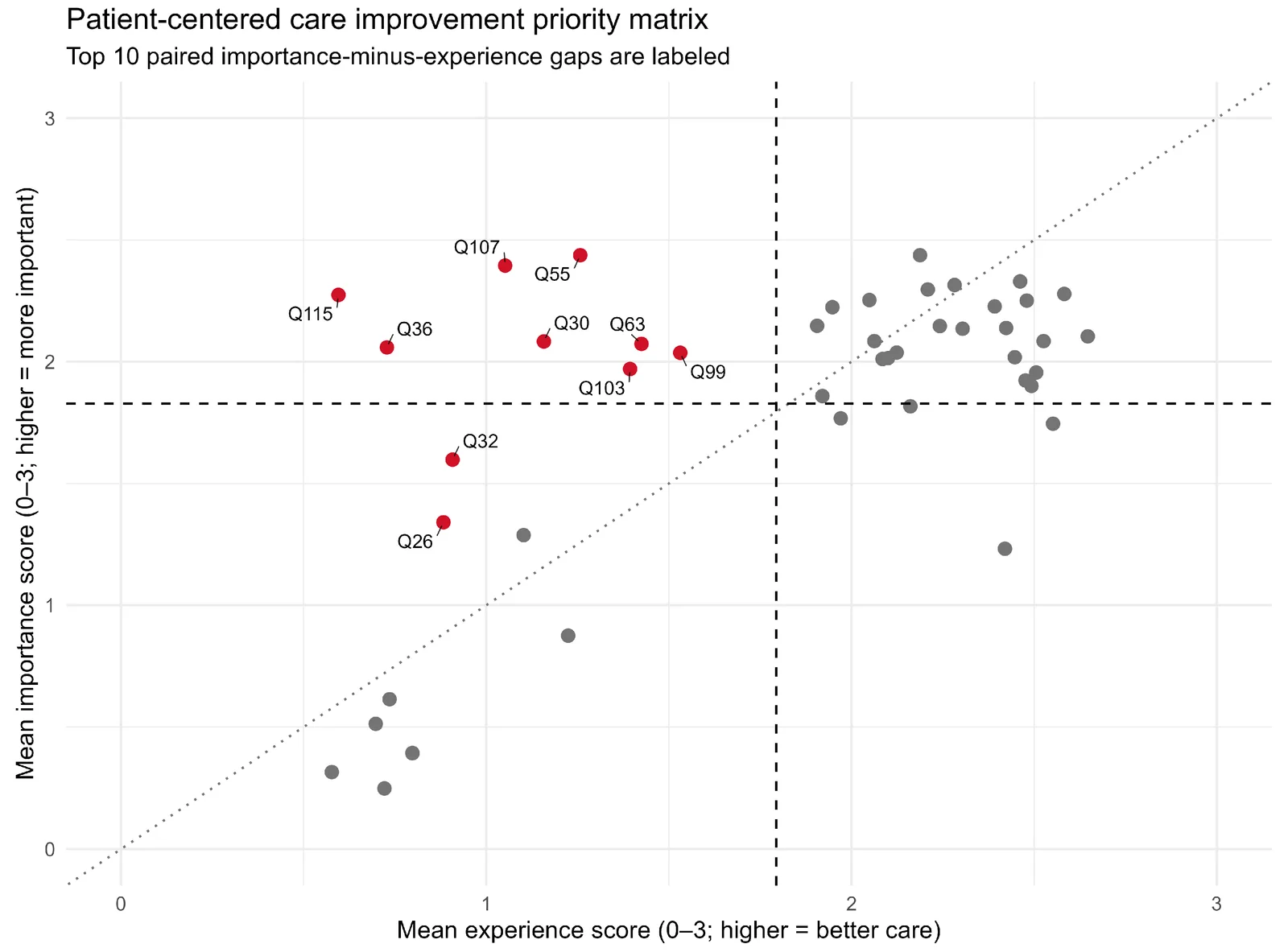
Patient-perceived priorities for improving fertility care; importance versus reported experience. Each point represents paired complete-case means for a patient-centered care item. Red points indicate the 10 items with the largest mean paired importance-minus-experience differences and are labeled with Q identifiers, whereas grey points indicate the remaining items. The dotted diagonal line represents equality between mean importance and experience scores. The vertical and horizontal dashed lines represent the respective mean values across all plotted items. Higher values on the *x*-axis indicate more favorable patient experience, and higher values on the *y*-axis indicate greater perceived importance. Items located toward the upper-left region represent potential quality-improvement priorities because they combine relatively high importance with comparatively poorer patient experience. Q labels correspond to questionnaire items listed in Supplementary Table S4.

The priority matrix placed these items according to their mean experience and importance scores, allowing high-importance and comparatively low-experience items to be distinguished visually from care aspects that were either less important or already experienced more favorably. This analysis was exploratory and was intended to support service-improvement prioritization rather than define a new psychometric subscale (Figure 2).

### 3.5. Global Care Ratings Showed a Strong Ceiling and Limited Explanatory Separation in Hierarchical Regression

Global fertility-care ratings were concentrated at the upper end of the response scale, with a mean of 9.59, a median of 10, and 79.1% of patients selecting 10; the distribution and residual diagnostics are shown in Supplementary Figures S1 and S2. In the common complete-case sample of 253 patients, the demographic model explained little variation in the global rating (R^2^ = 0.018; adjusted R^2^ = 0.002). None of the demographic coefficients was statistically significant using HC3 robust inference, including age category (B = 0.134, 95% CI −0.094 to 0.361; *p* = 0.247), education, or clinic affiliation (Table 4).

Adding the patient-centered care domains increased R^2^ to 0.117 and adjusted R^2^ to 0.065, corresponding to a change in R^2^ of 0.099. The conventional nested-model test indicated an incremental contribution of the added domains, F-change(10, 238) = 2.67, *p* = 0.004. However, the HC3 robust joint Wald test for the same block did not reach the conventional 0.05 threshold, F(10, 238) = 1.71, *p* = 0.080, indicating that the overall block-level inference was sensitive to the variance estimator (Table 4).

Within Model 2, three domain coefficients reached nominal significance with HC3 inference. Minimizing Cost was inversely associated with the global rating (B = −0.150, 95% CI −0.275 to −0.026; *p* = 0.018), whereas the Physical Comfort proxy (B = 0.186, 95% CI 0.026 to 0.345; *p* = 0.023) and Emotional and Psychological Support (B = 0.337, 95% CI 0.057 to 0.617; *p* = 0.019) were positively associated with the global rating. Accessibility showed a positive but nonsignificant coefficient (B = 0.303, *p* = 0.122), and all remaining demographic and domain terms had *p* values above 0.05 (Table 4). Given the nonsignificant robust block test and the instability of individual coefficients in sensitivity analyses, all regression coefficients were interpreted as exploratory associations.

### 3.6. Sensitivity Analyses Supported the Major Perception Gaps but Not a Stable Set of Satisfaction Predictors

After retaining the first occurrence of each exact patient response pattern, the major patient–staff contrasts remained directionally consistent. Minimizing Cost remained strongly higher among staff (difference 1.189, 95% CI 0.953 to 1.426; *p* < 0.001), as did Staff Attitude and Communication (1.196, 95% CI 1.109 to 1.284; *p* < 0.001), Information and Education (1.081, 95% CI 0.942 to 1.220; *p* < 0.001), and Participation in Care (0.542, 95% CI 0.404 to 0.680; *p* < 0.001). Privacy, Emotional and Psychological Support, and Continuity and Coordination continued to favor patient ratings. Accessibility and Staff Competence remained nonsignificant, while the Physical Comfort proxy decreased to a difference of 0.318 and was no longer statistically significant (FDR *p* = 0.105) (Supplementary Table S5). These findings indicate that the principal group-level contrast directions were not created by the repeated exact patterns, although uncertainty increased after the restriction and the Physical Comfort proxy was not robust.

The one-pattern-per-record regression was less supportive of the individual full-data coefficients. Minimizing Cost (B = −0.146, *p* = 0.156) and the Physical Comfort proxy (B = 0.138, *p* = 0.233) were no longer significant, whereas Emotional and Psychological Support remained positively associated with the global rating (B = 0.368, 95% CI 0.005 to 0.732; *p* = 0.047). The other demographic and domain coefficients remained nonsignificant (Supplementary Table S6).

The HC3 top-box logistic sensitivity model included 207 ratings of 10 and 46 ratings below 10 with 14 non-intercept predictor parameters. Postgraduate or other education relative to secondary or vocational education was associated with lower odds of a 10/10 rating (OR = 0.15, 95% CI 0.03 to 0.71; *p* = 0.017), while the Physical Comfort proxy was associated with higher odds (OR = 1.72, 95% CI 1.12 to 2.65; *p* = 0.014). Age category (OR = 1.71, 95% CI 0.99 to 2.97; *p* = 0.055), Staff Attitude and Communication (OR = 2.71, 95% CI 0.96 to 7.63; *p* = 0.059), and Participation in Care (OR = 0.42, 95% CI 0.16 to 1.08; *p* = 0.072) did not meet the nominal 0.05 threshold. Because only 46 observations were in the lower-frequency outcome group, these estimates were treated as exploratory and not as stable independent predictors (Supplementary Table S7).

## 4. Discussion

### 4.1. Principal Findings and the Direction of Group-Level Patient–Staff Differences

The present analysis shows that disagreement between fertility-care staff and patients was substantial, but it was not uniformly in the direction of staff rating care more favorably. Staff scores were markedly higher for Minimizing Cost, Staff Attitude and Communication, Information and Education, and Participation in Care, while patients gave more favorable ratings for Privacy, Emotional and Psychological Support, and Continuity and Coordination. Accessibility and Staff Competence showed little evidence of a between-group difference. This bidirectional pattern is important because it suggests that provider self-assessment cannot be used as a simple surrogate for patient-reported experience, even when both groups are evaluating the same prespecified service indices.

The clinic-stratified findings also indicate that the major cost, communication, information, privacy, continuity, and participation gaps were not produced solely by combining respondents from different clinics. However, the Physical Comfort proxy and emotional-support differences were evident in Persona but not IRM. With only two clinics and all repeated exact patterns concentrated in IRM, these results cannot separate respondent-level perceptions from clinic-specific organizational context and should not be interpreted as causal clinic effects.

Previous fertility-care research has repeatedly shown that professionals and patients do not necessarily assign the same value to patient-centered aspects of care. In a discrete-choice study, physicians placed less relative importance on patient-centered attributes than patients did, demonstrating that professional priorities may diverge from what fertility patients value [32].

The direction of misperception can also vary across settings. A Swedish multicenter IVF study found that healthcare professionals generally underestimated patients’ satisfaction across patient-centered quality dimensions, whereas our data contained both staff-favorable and patient-favorable gaps [33].

The large differences in Minimizing Cost, Information and Education, and Participation in Care are consistent with the broader fertility-care literature showing that patients place high value on accessible information, involvement, coordination, support, and respectful relationships with clinic staff [34].

These findings are also compatible with qualitative work among Arab women with infertility, in which Accessibility, Minimizing Cost, Information and Education, and Staff Attitude and Communication were among the dimensions most consistently emphasized by participants [12].

The cost gap should not be interpreted as direct evidence that a particular clinic was unaffordable, because the domain reflects patient and staff perceptions rather than verified expenditure. It nevertheless identifies financial experience as an area in which staff assessments were considerably more favorable than patient reports. This deserves attention because out-of-pocket ART expenditures can be substantial and financially burdensome in low- and middle-income settings [35].

The patient-favorable differences in Continuity and Coordination, Privacy, and Emotional and Psychological Support are equally informative. In the original Dutch PCQ-Infertility validation, continuity was among the least favorably rated patient-centered dimensions, whereas patients in the present study rated continuity relatively highly [11].

Rather than assuming that one direction is intrinsically correct, these discrepancies should be understood as evidence that staff and patients may use different reference standards when evaluating the same care process. Fertility-care quality improvement therefore benefits from collecting patient-reported experience directly and interpreting it alongside, rather than replacing it with, professional self-assessment.

### 4.2. Measurement Performance and Patient-Defined Priorities for Improvement

The internal-consistency and item-diagnostic findings require a more cautious interpretation of the preliminarily adapted survey than was possible from the earlier analysis. Reliability was heterogeneous across domains, and several alpha coefficients were low. The negative patient alpha for Participation in Care is particularly important because it indicates that the included items did not behave as a coherent additive scale under the present scoring and sample conditions. The negative inter-item and corrected item-total correlations in Participation in Care show that these items did not form a coherent additive index under the implemented scoring. Reverse-coded rules were rechecked in the reproducible mapping, but definitive scoring verification was not possible without the administered item-level codebook; the index should therefore not be used as a stand-alone scale.

McDonald omega provided additional information, but higher omega values in some domains did not remove the need to examine dimensionality and item behavior. Methodological work has emphasized that alpha is often used under assumptions that are not met in practice, while omega can be preferable when scale items have unequal relationships with the underlying construct. Neither coefficient, however, establishes structural validity on its own [25,26].

This distinction is particularly relevant for Staff Continuity and Coordination, where alpha was 0.213 but omega was 0.729. Such divergence should not be summarized as evidence of satisfactory reliability without examining factor structure, sparse response categories, and the effect of missing data. Contemporary COSMIN guidance similarly separates internal consistency from evidence that the items form the intended unidimensional construct [18].

The importance–experience analysis adds a different and practically useful perspective because it does not assume that every care item should contribute equally to a latent scale. The largest gaps involved privacy, cost, information provision, participation, emotional support, accessibility, and waiting or comfort. These dimensions closely resemble the care needs identified in systematic and qualitative fertility-care research, where patients consistently emphasized information, coordination, accessibility, emotional support, privacy, partner involvement, and respectful interaction [34,36].

The priority matrix should therefore be interpreted as a service-improvement tool rather than a validation analysis. The original PCQ-Infertility also paired patient experience with importance ratings so that poor performance on highly valued aspects of care could be distinguished from poor performance on less important aspects [11].

This distinction is useful for practice. For example, a clinic may have a modest absolute score on one item, but if patients consider that item highly important, it can reasonably receive greater attention in improvement planning. Positive patient-centered experiences have also been associated with stronger intentions to comply with fertility treatment, supporting the relevance of patient experience for service engagement even when clinical outcomes are not measured directly [37].

### 4.3. Global Care Ratings, Ceiling Effects, and the Instability of Predictor Models

The global care rating was extremely favorable, with a median of 10 and almost four in five patients selecting the highest possible score. Such a ceiling restricts outcome variability and makes it difficult for a global rating to discriminate between otherwise meaningful differences in care experience. Ceiling effects are common in patient-satisfaction and patient-experience measures and can reduce their sensitivity for quality-improvement and explanatory analyses [38].

This distribution helps explain why the multivariable findings should be interpreted conservatively. In the common complete-case sample, demographic variables explained very little variation in global ratings. Adding the care domains increased conventional R-squared by approximately ten percentage points, and the conventional F-change test was significant, but the HC3 robust joint test of the added domain block was not. The difference between these tests indicates that the global conclusion about incremental explanatory value depends on how sampling variability is estimated.

At the coefficient level, Minimizing Cost, the Physical Comfort proxy, and Emotional and Psychological Support reached nominal significance in the full-data robust linear model. However, only Emotional and Psychological Support remained nominally significant in the one-pattern-per-record sensitivity analysis, and the top-box logistic model identified a different combination of associations. These results do not support describing any single domain, demographic characteristic, or clinic as a definitive independent driver of satisfaction.

The finding that communication-related measures did not produce a stable linear association with global satisfaction is not necessarily inconsistent with the broader literature. In an Italian multicenter study of ART consultations, patients reported high satisfaction, but the observed patient-centered communication index was not associated with satisfaction or retention in care, illustrating that global satisfaction can be influenced by factors that are not captured by a single communication metric [8].

The current findings therefore favor domain-specific patient experience measures over reliance on a single global satisfaction score. Global ratings remain useful as summary indicators, but when they are highly concentrated at the maximum value, they provide limited resolution for identifying which aspects of care should change.

### 4.4. Implications, Strengths, Limitations, and Priorities for Further Research

The practical implication of the patient–staff contrasts is not that staff judgments are uniformly inaccurate, but that routine quality assessment should include both perspectives and should focus on domains where the difference is large enough to be operationally meaningful. The strongest staff-favorable gaps in cost, information, communication, and participation suggest that clinics should examine whether existing information channels, explanations of financial obligations, opportunities for questions, and shared decision processes are experienced by patients as intended.

Patient feedback alone may not be sufficient to change practice. A mixed-method fertility-care study found that audit and feedback to professionals did not automatically produce broad improvements in patient-centeredness, indicating that feedback is more likely to be useful when it is linked to specific organizational and behavioral interventions [39].

The present study has several strengths. Patient and staff responses were analyzed using an explicit common scoring direction, comparisons were restricted to semantically matched care content, effect sizes and confidence intervals were reported alongside multiplicity-adjusted *p* values, and the regression analysis used a common complete-case sample with heteroskedasticity-robust inference. The analysis also incorporated patient importance ratings and repeated the main comparisons after retaining one record per unique exact response pattern, which made it possible to distinguish stable perception gaps from unstable satisfaction-model coefficients.

Several limitations remain. First, the data came from only two clinics in one city; clinic-level clustering could not be modeled reliably, and the findings are not national estimates of fertility-care quality in Kazakhstan. Second, the archived materials did not document the recruitment approach, numbers approached or declining, response-rate denominator, questionnaire administration mode, or data-entry quality-control workflow. Selection and data-entry bias therefore cannot be quantified. Third, patient and staff respondents were independent rather than paired at the provider–patient level, and semantic matching was based on item content rather than formal measurement-invariance testing. Fourth, the adaptation records did not preserve translator qualifications, independence, reconciliation logs, or item-specific cognitive interview changes, and no Kazakhstan-specific quantitative expert-rating dataset was available. The process should therefore be understood as preliminary cultural and linguistic adaptation rather than validation.

Fifth, the heterogeneous response formats required item-specific harmonization, the administered codebook was unavailable, 308 item responses fell outside the implemented scoring sets, and several computed scores disagreed with legacy conversion columns. Of these unexpected item responses, 240 occurred in patient importance fields, including 206 in P39, which was not included in the paired priority analysis because the corresponding experience item was unscored. The scoring-dependent results are therefore conditional on the transparent mapping supplied in Supplementary Code S1. The Physical Comfort proxy was a single waiting-room item, several multi-item indices had weak or negative internal consistency, and structural validity, test–retest reliability, measurement invariance, and responsiveness were not evaluated. Sixth, the global rating had an extreme ceiling and the top-box model had only 46 below-top-box observations for 14 predictor parameters, so all regression results are exploratory. Finally, all 100 records beyond the first occurrence of an exact pattern were concentrated in IRM. Because identifiers, timestamps, and linkable source records were unavailable, these patterns could not be verified or classified as duplicate participants. The principal perception-gap directions were generally preserved in one-pattern-per-record analyses, but the effective sample size remains uncertain and the satisfaction-model coefficients were unstable.

Future work should therefore use a larger multicenter sample, verify the source codebook and finalize the item-index mapping before field administration, evaluate structural validity with methods appropriate for ordinal survey data, examine test–retest reliability, and test whether the measurement structure is comparable across language, clinic, and respondent groups. The original PCIQ-F development process itself anticipated additional psychometric testing after content development, and more recent revisions of infertility patient-centeredness instruments similarly emphasize continued measurement refinement rather than a single validation step [1,40].

Longitudinal research should also investigate whether the domains identified as patient priorities are associated with treatment continuation, psychological burden, and care engagement. Organizational and psychological burden are established reasons for discontinuation of fertility treatment, which makes patient-centered service design relevant beyond satisfaction alone [41].

## 5. Conclusions

This study provides an exploratory survey-based assessment of patient-centered fertility care after preliminary cultural and linguistic adaptation in two Almaty ART clinics. The strongest finding was not a universal tendency for staff to overestimate care, but a domain-specific and bidirectional pattern of disagreement. Staff rated cost, communication, information, and participation more favorably, whereas patients rated privacy, emotional support, and continuity more favorably. The paired patient-defined priority analysis identified concrete targets for service review, particularly in privacy, cost, information, and participation.

At the same time, the heterogeneous internal-consistency estimates, the strong ceiling in global care ratings, and the sensitivity of regression coefficients show that the questionnaire should currently be regarded as an exploratory survey for preliminary quality-improvement assessment, not as an established psychometric instrument. Source-codebook confirmation, broader multicenter testing, and structural and longitudinal validation are required before national benchmarking or strong predictive claims are justified.

## Figures and Tables

**Table 1:** Participant characteristics of patients and fertility-care staff in two Almaty ART clinics.

Characteristic		*n*	%
Patient (*n* = 273)			
Clinic (*n* = 272)	Persona	145	53.3
	IRM	127	46.7
Education	Secondary/vocational secondary	75	27.5
	Higher professional/university	156	57.1
	Postgraduate education	38	13.9
	Other	4	1.5
Current/recent treatment	Treatment not started	22	8.1
	IVF/ICSI	228	83.5
	Other ART/treatment	23	8.4
Age category	<20 years	16	5.9
	20–24 years	48	17.6
	25–29 years	65	23.8
	30–34 years	142	52.0
	35–39 years	2	0.7
Ethnicity	Kazakh	176	64.5
	Other	95	34.8
	Other/unknown code	2	0.7
Previous pregnancy	Yes, without fertility treatment	108	39.6
	Yes, after fertility treatment	56	20.5
	Yes, both with and without treatment	21	7.7
	No	88	32.2
Infertility cause	Male factor	49	17.9
	Female factor	142	52.0
	Both male and female	23	8.4
	Unexplained	51	18.7
	Don’t know	8	2.9
Staff (*n* = 74)			
Clinic	Persona	43	58.1
	IRM	31	41.9
Education	Secondary/vocational secondary	27	36.5
	Higher professional/university	42	56.8
	Postgraduate education	5	6.8
Profession	Doctor	30	40.5
	Embryologist	7	9.5
	Nurse	28	37.8
	Laboratory technician	7	9.5
	Other	2	2.7
Age category	<20 years	7	9.5
	20–24 years	12	16.2
	25–29 years	7	9.5
	30–34 years	19	25.7
	35–39 years	7	9.5
	>40 years	22	29.7
Gender	Female	62	83.8
	Male	12	16.2
Work experience	<1 year	7	9.5
	1–5 years	12	16.2
	6–10 years	18	24.3
	11–20 years	19	25.7
	>20 years	18	24.3

**Table 2:** Patient–staff differences at the group level in prespecified matched patient-centered fertility-care indices.

Domain	Patient *n*	Patient Mean ± SD	Staff *n*	Staff Mean ± SD	Staff–Patient Difference	95% CI	Hedges g	FDR *p*
Accessibility	269	2.147 ± 0.515	74	2.197 ± 0.397	0.050	−0.061 to 0.160	0.101	0.418
Minimizing Cost	273	1.049 ± 0.897	74	2.291 ± 0.832	1.241	1.022 to 1.461	1.401	<0.001
Physical Comfort proxy	273	1.531 ± 1.141	53	1.925 ± 1.174	0.393	0.044 to 0.743	0.342	0.035
Privacy	254	1.937 ± 0.626	70	1.538 ± 0.496	−0.399	−0.539 to −0.258	−0.662	<0.001
Staff Attitude and Communication	273	1.020 ± 0.486	74	2.150 ± 0.258	1.130	1.047 to 1.213	2.520	<0.001
Staff Competence	273	2.401 ± 0.587	74	2.385 ± 0.457	−0.016	−0.142 to 0.110	−0.028	0.803
Information and Education	273	1.329 ± 0.515	74	2.310 ± 0.500	0.981	0.851 to 1.112	1.914	<0.001
Emotional and Psychological Support	273	2.037 ± 0.513	74	1.809 ± 0.455	−0.228	−0.350 to −0.107	−0.455	<0.001
Continuity and Coordination	273	2.285 ± 0.584	74	1.457 ± 0.427	−0.829	−0.949 to −0.708	−1.492	<0.001
Participation in Care	273	1.647 ± 0.435	74	2.225 ± 0.524	0.579	0.447 to 0.710	1.268	<0.001

Note: Scores were harmonized to 0–3, with higher scores indicating more favorable patient-centered care. Groups are independent rather than paired. Differences are staff minus patient scores. Welch independent-samples tests were used; *p* values are Benjamini–Hochberg FDR-adjusted. The indices are prespecified descriptive composites and should not be interpreted as validated unidimensional scales. Hedges’ g is reported as the standardized mean difference.

**Table 3:** Internal consistency of multi-item patient-centered care domains.

Group	Domain	Items	Complete Cases	Cronbach α	Standardized α	McDonald ω
Patient	Accessibility	5	216	0.252	0.259	0.307
	Privacy	4	151	0.204	0.325	0.564
	Staff Attitude and Communication	7	236	0.605	0.597	0.784
	Staff Competence	4	273	0.393	0.389	0.495
	Information and Education	9	235	0.591	0.650	0.787
	Emotional and Psychological Support	7	94	0.441	0.478	0.647
	Continuity and Coordination	8	273	0.647	0.663	0.674
	Participation in Care	3	272	−0.238	−0.235	0.277
Staff	Accessibility	4	63	0.157	0.102	0.349
	Privacy	4	16	0.175	0.207	0.386
	Staff Attitude and Communication	6	73	0.375	0.461	0.485
	Staff Competence	4	74	0.299	0.272	0.427
	Information and Education	9	63	0.615	0.643	0.652
	Emotional and Psychological Support	7	30	0.547	0.555	0.633
	Continuity and Coordination	8	55	0.213	0.158	0.729
	Participation in Care	3	74	0.304	0.319	0.387

Note: Reliability coefficients are descriptive preliminary estimates only. Single-item/proxy constructs are not included. Low or negative α values indicate that some item sets should not presently be interpreted as established reflective scales. ω should not be used to override clearly problematic item behavior without further structural analysis.

**Table 4:** Hierarchical regression of patients’ global fertility-care ratings with HC3 robust inference.

Model	Predictor	Unstandardized B	HC3 SE	95% CI	*p*
Model 1: Demographic characteristics	Age category, per category	0.134	0.115	−0.094 to 0.361	0.247
	Higher professional/university vs. secondary/vocational	−0.047	0.172	−0.387 to 0.292	0.784
	Postgraduate/other vs. secondary/vocational	−0.172	0.167	−0.500 to 0.156	0.302
	Persona vs. IRM	−0.108	0.124	−0.353 to 0.138	0.389
	Model 1 summary	*n* = 253; R^2^ = 0.018; adjusted R^2^ = 0.002			
Model 2: Demographic characteristics + PCC domains	Age category, per category	0.030	0.107	−0.180 to 0.241	0.776
	Higher professional/university vs. secondary/vocational	0.017	0.181	−0.340 to 0.374	0.925
	Postgraduate/other vs. secondary/vocational	0.013	0.200	−0.381 to 0.407	0.949
	Persona vs. IRM	−0.045	0.164	−0.368 to 0.277	0.781
	Accessibility	0.303	0.195	−0.081 to 0.688	0.122
	Minimizing Cost	−0.150	0.063	−0.275 to −0.026	0.018
	Physical Comfort proxy	0.186	0.081	0.026 to 0.345	0.023
	Privacy	0.008	0.167	−0.321 to 0.336	0.963
	Staff Attitude and Communication	−0.108	0.230	−0.561 to 0.345	0.638
	Staff Competence	−0.010	0.188	−0.380 to 0.361	0.959
	Information and Education	0.010	0.129	−0.245 to 0.264	0.940
	Emotional and Psychological Support	0.337	0.142	0.057 to 0.617	0.019
	Continuity and Coordination	−0.148	0.155	−0.454 to 0.158	0.341
	Participation in Care	−0.042	0.148	−0.333 to 0.250	0.778
	Model 2 summary	*n* = 253; R^2^ = 0.117; adjusted R^2^ = 0.065; ΔR^2^ = 0.099; conventional F-change(10, 238) = 2.67, *p* = 0.004; HC3 robust Wald F(10, 238) = 1.71, *p* = 0.080			

Note: Both models were estimated using the same complete-case sample (*n* = 253). Model 1 included age category, education, and clinic affiliation; Model 2 additionally included patient-centered care-domain scores. B represents the unstandardized regression coefficient. Standard errors, 95% confidence intervals, and coefficient *p*-values are based on HC3 heteroskedasticity-robust variance estimates. The reference categories were secondary/vocational secondary education and IRM clinic. The conventional nested-model F-change is reported together with an HC3 robust Wald test of the additional PCC-domain block. “Physical Comfort proxy” represents the available single-item waiting/comfort indicator rather than a validated multi-item Physical Comfort scale.

## Data Availability

To protect participants’ privacy and confidentiality, the datasets are not publicly available. However, the datasets used and/or analyzed during the current study are available from the corresponding authors on reasonable request.

## Supplementary Materials

The following supporting information can be downloaded at: https://www.mdpi.com/article/10.3390/healthcare14182941/s1, Table S1: R software environment and packages used for data management, statistical analysis, and graphical presentation; Table S2: Descriptive statistics and floor/ceiling effects for domain scores; Table S3: Data-quality, scoring, and outcome-distribution audit; Table S4: Paired patient importance-experience differences; Table S5: One-pattern-per-record sensitivity analysis of patient-staff differences; Table S6: Exact-pattern sensitivity analysis of the global-rating regression; Table S7: Top-box logistic sensitivity analysis with HC3 robust inference; Table S8: Clinic-stratified patient-staff comparisons in submitted-record and one-pattern-per-record analyses; Table S9: Item-level patient-staff comparisons in submitted-record and one-pattern-per-record analyses; Table S10: Item diagnostics for prespecified indices with very low or negative Cronbach alpha; Table S11: Pairwise inter-item correlations for prespecified indices with very low or negative Cronbach alpha; Table S12: Item-level scoring crosswalk, unexpected-code handling, and scoring reconciliation; Figure S1: Distribution of the patient global fertility-care rating. The maximum rating of 10 accounted for 79.1% of patient records; Figure S2: Exploratory linear-model residual diagnostics. The bounded ceiling-skewed outcome limits the interpretation of ordinary least-squares residual diagnostics and coefficients; Supplementary Code S1: R code for data processing, scoring, statistical analyses, sensitivity analyses, and generation of tables and figures.

## Abbreviations

The following abbreviations are used in this manuscript:

PCC	Patient-centered care
ART	Assisted reproductive technology
IVF	In vitro fertilization
ICSI	Intracytoplasmic sperm injection
PCIQ-F	Patient-Centered Infertility Questionnaire for Female Clients
PCQ-Infertility	Patient-Centeredness Questionnaire for Infertility
IRM	Institute of Reproductive Medicine
STROBE	Strengthening the Reporting of Observational Studies in Epidemiology
COSMIN	COnsensus-based Standards for the selection of health Measurement INstruments
HC3	Heteroskedasticity-consistent covariance estimator, type 3
FDR	False discovery rate
CI	Confidence interval
SD	Standard deviation
SE	Standard error
IQR	Interquartile range
OR	Odds ratio
R^2^	Coefficient of determination

## References

[1] Webair H.H., Ismail T.A.T., Ismail S.B., Khaffaji A.J., Hussain N.H.N., Kadir A.A., Muhamad R., Abu Bakar F.A., Ismail N.R., Badri N. (2021). Patient-centered infertility questionnaire for female clients (PCIQ-F): Part I: Questionnaire development. BMC Med. Res. Methodol..

[2] Committee on Quality of Health Care in America, Institute of Medicine (2001). Crossing the Quality Chasm: A New Health System for the 21st Century.

[3] Epstein R.M., Street R.L. (2011). The values and value of patient-centered care. Ann. Fam. Med..

[4] Huppelschoten A.G., van Duijnhoven N.T., Hermens R.P., Verhaak C., Kremer J.A., Nelen W.L.D.M. (2012). Improving patient-centeredness of fertility care using a multifaceted approach: Study protocol for a randomized controlled trial. Trials.

[5] Vander Borght M., Wyns C. (2018). Fertility and infertility: Definition and epidemiology. Clin. Biochem..

[6] Thoma M., Fledderjohann J., Cox C., Kantum Adageba R. (2021). Biological and Social Aspects of Human Infertility: A Global Perspective.

[7] Nicoloro-SantaBarbara J., Busso C., Moyer A., Lobel M. (2018). Just relax and you’ll get pregnant? Meta-analysis examining women’s emotional distress and the outcome of assisted reproductive technology. Soc. Sci. Med..

[8] Borghi L., Leone D., Poli S., Becattini C., Chelo E., Costa M., De Lauretis L., Ferraretti A.P., Filippini C., Giuffrida G. (2019). Patient-centered communication, patient satisfaction, and retention in care in assisted reproductive technology visits. J. Assist. Reprod. Genet..

[9] Ebrahimzadeh Zagami S., Latifnejad Roudsari R., Janghorban R., Mousavi Bazaz S.M., Amirian M., Allan H.T. (2019). A qualitative study of the challenges experienced by Iranian infertile couples after unsuccessful assisted reproductive technologies. Int. J. Women’s Health Reprod. Sci..

[10] Zurlo M.C., Cattaneo Della Volta M.F., Vallone F. (2019). The association between stressful life events and perceived quality of life among women attending infertility treatments: The moderating role of coping strategies and perceived couple’s dyadic adjustment. BMC Public Health.

[11] van Empel I.W.H., Aarts J.W.M., Cohlen B.J., Huppelschoten D.A., Laven J.S.E., Nelen W.L.D.M., Kremer J.A.M. (2010). Measuring patient-centredness, the neglected outcome in fertility care: A random multicentre validation study. Hum. Reprod..

[12] Webair H.H., Ismail T.A.T., Ismail S.B., Khaffaji A.J. (2021). Patient-centred infertility care among Arab women experiencing infertility: A qualitative study. BMJ Open.

[13] Dancet E.A.F., van Empel I.W.H., Rober P., Nelen W.L.D.M., Kremer J.A.M., D’Hooghe T.M. (2011). Patient-centred infertility care: A qualitative study to listen to the patient’s voice. Hum. Reprod..

[14] Liang J., Jie Q., Xu W., Li J., Fu M., Liu P., Chen Y., Wang X., Li X., Li Z. (2025). The difference in patient-centered medical experiences between public fertility care and private fertility care in China: A multicenter cross-sectional study. BMC Public Health.

[15] Issanov A., Aimagambetova G., Terzic S., Bapayeva G., Ukybassova T., Baikoshkarova S., Utepova G., Daribay Z., Bekbossinova G., Balykov A. (2022). Impact of governmental support on IVF clinical pregnancy rates: Differences between public and private clinical settings in Kazakhstan, a prospective cohort study. BMJ Open.

[16] Lokshin V., Omar M., Karibaeva S. (2022). Assisted reproductive technologies in the Republic of Kazakhstan: A 6-year trend analysis from efficacy to availability. J. Reprod. Infertil..

[17] Suleimenova M., Lokshin V., Glushkova N., Karibayeva S., Terzic M. (2022). Quality-of-life assessment of women undergoing in vitro fertilization in Kazakhstan. Int. J. Environ. Res. Public Health.

[18] Terwee C.B., Prinsen C.A.C., Chiarotto A., Westerman M.J., Patrick D.L., Alonso J., Bouter L.M., de Vet H.C.W., Mokkink L.B. (2018). COSMIN methodology for evaluating the content validity of patient-reported outcome measures: A Delphi study. Qual. Life Res..

[19] von Elm E., Altman D.G., Egger M., Pocock S.J., Gotzsche P.C., Vandenbroucke J.P. (2007). The Strengthening the Reporting of Observational Studies in Epidemiology (STROBE) statement: Guidelines for reporting observational studies. Lancet.

[20] Sharma A., Minh Duc N.T., Luu Lam Thang T., Nam N.H., Ng S.J., Abbas K.S., Huy N.T., Marušić A., Paul C.L., Kwok J. (2021). A Consensus-Based Checklist for Reporting of Survey Studies (CROSS). J. Gen. Intern. Med..

[21] Beaton D.E., Bombardier C., Guillemin F., Ferraz M.B. (2000). Guidelines for the process of cross-cultural adaptation of self-report measures. Spine.

[22] Sousa V.D., Rojjanasrirat W. (2011). Translation, adaptation and validation of instruments or scales for use in cross-cultural health care research: A clear and user-friendly guideline. J. Eval. Clin. Pract..

[23] Boateng G.O., Neilands T.B., Frongillo E.A., Melgar-Quinonez H.R., Young S.L. (2018). Best practices for developing and validating scales for health, social, and behavioral research: A primer. Front. Public Health.

[24] R Core Team (2024). R: A Language and Environment for Statistical Computing.

[25] Hayes A.F., Coutts J.J. (2020). Use omega rather than Cronbach’s alpha for estimating reliability. But…. Commun. Methods Meas..

[26] McNeish D. (2018). Thanks coefficient alpha, we’ll take it from here. Psychol. Methods.

[27] Welch B.L. (1947). The generalization of Student’s problem when several different population variances are involved. Biometrika.

[28] Hedges L.V. (1981). Distribution theory for Glass’s estimator of effect size and related estimators. J. Educ. Stat..

[29] Benjamini Y., Hochberg Y. (1995). Controlling the false discovery rate: A practical and powerful approach to multiple testing. J. R. Stat. Soc. Ser. B.

[30] MacKinnon J.G., White H. (1985). Some heteroskedasticity-consistent covariance matrix estimators with improved finite sample properties. J. Econom..

[31] Zeileis A. (2004). Econometric computing with HC and HAC covariance matrix estimators. J. Stat. Softw..

[32] van Empel I.W.H., Dancet E.A.F., Koolman X.H.E., Nelen W.L.D.M., Stolk E.A., Sermeus W., D’Hooghe T.M., Kremer J.A.M. (2011). Physicians underestimate the importance of patient-centredness to patients: A discrete choice experiment in fertility care. Hum. Reprod..

[33] Holter H., Gejervall A.L., Borg K., Sandin-Bojo A.K., Wikland M., Wilde-Larsson B., Bergh C. (2017). In vitro fertilization healthcare professionals generally underestimate patients’ satisfaction with quality of care. Acta Obstet. Gynecol. Scand..

[34] Dancet E.A.F., Nelen W.L.D.M., Sermeus W., De Leeuw L., Kremer J.A.M., D’Hooghe T.M. (2010). The patients’ perspective on fertility care: A systematic review. Hum. Reprod. Update.

[35] Njagi P., Groot W., Arsenijevic J., Dyer S., Mburu G., Kiarie J. (2023). Financial costs of assisted reproductive technology for patients in low- and middle-income countries: A systematic review. Hum. Reprod. Open.

[36] Dancet E.A.F., D’Hooghe T.M., van der Veen F., Bossuyt P., Sermeus W., Mol B.W., Repping S. (2014). “Patient-centered fertility treatment”: What is required?. Fertil. Steril..

[37] Pedro J., Canavarro M.C., Boivin J., Gameiro S. (2013). Positive experiences of patient-centred care are associated with intentions to comply with fertility treatment: Findings from the validation of the Portuguese version of the PCQ-Infertility tool. Hum. Reprod..

[38] Voutilainen A., Pitkaaho T., Kvist T., Vehvilainen-Julkunen K. (2016). How to ask about patient satisfaction? The visual analogue scale is less vulnerable to confounding factors and ceiling effect than a symmetric Likert scale. J. Adv. Nurs..

[39] Huppelschoten A.G., Aarts J.W.M., van Empel I.W.H., Cohlen B.J., Kremer J.A.M., Nelen W.L.D.M. (2013). Feedback to professionals on patient-centered fertility care is insufficient for improvement: A mixed-method study. Fertil. Steril..

[40] van der Kolk L., Smit E., Bloemer J., van Wijk L.M. (2023). The PCQ-Infertility Revised: A new digital instrument to measure treatment satisfaction of fertility patients. Patient Relat. Outcome Meas..

[41] Gameiro S., Boivin J., Peronace L., Verhaak C.M. (2012). Why do patients discontinue fertility treatment? A systematic review of reasons and predictors of discontinuation in fertility treatment. Hum. Reprod. Update.

