# Supplementary material for: Patient-Centered Fertility Care in Kazakhstan: Preliminary Cultural Adaptation of a Fertility-Care Survey and Patient–Staff Perception Gaps in Two Assisted-Reproduction Clinics
Source: Healthcare (Basel). 2026 Sep 10;14(18):2941. doi: 10.3390/healthcare14182941 (PMC13606665; doi:10.3390/healthcare14182941)
Supplement: Supplementary file 1 [file healthcare-14-02941-s001.zip › healthcare-4520377-supplementary.pdf]

Supplementary tables

Table S1. R software environment and packages used for data management, statistical analysis, and graphical presentation.

Software/package	Version	Role in the analysis
R	4.3.3	Statistical computing environment used for data processing, statistical analyses, sensitivity analyses, and graphical output
readxl	1.4.5	Import of patient, staff, questionnaire-mapping, and supporting data from Excel workbooks
dplyr	1.1.4	Data cleaning, recoding, filtering, construction of domain scores, grouping, and descriptive summaries
tidyr	1.3.1	Data restructuring and construction of complete-case analysis datasets
purrr	1.0.4	Repeated application of scoring and analytical functions across items and domains
stringr	1.6.0	Processing and management of questionnaire-item names and text mappings
psych	2.5.6	Estimation of Cronbach's alpha, standardized alpha, and McDonald's omega
sandwich	3.1-3	Estimation of HC3 heteroskedasticity-consistent covariance matrices
lmtest	0.9-40	Robust coefficient testing and model-related inferential procedures
broom	1.0.13	Conversion of fitted statistical models into structured tabular outputs
MASS	7.3-60.0.1	Statistical modeling functions used in supplementary analytical procedures
ggplot2	3.5.2	Generation of publication-quality statistical figures
ggrepel	0.9.6	Placement of non-overlapping questionnaire-item labels in the importance-experience priority matrix
ragg	1.3.3	High-resolution rendering of publication figures
readr	2.1.5	Export and management of analysis results in delimited-text formats
writexl	1.5.3	Export of analysis results and manuscript tables to Excel workbooks

Table S2. Descriptive statistics and floor/ceiling effects for domain scores.

Group	Domain	n	Mean	SD	Median	IQR	Floor %	Ceiling %
Patient	Accessibility	273	2.146	0.481	2.150	1.800-2.550	0.0	0.0
	Minimizing Cost	273	1.049	0.897	1.500	0.000-1.500	37.4	7.3
	Physical Comfort proxy	273	1.531	1.141	2.000	0.000-3.000	27.1	25.3
	Privacy	254	1.937	0.626	2.000	1.667-2.250	0.0	7.1
	Staff Attitude and Communication	273	1.020	0.486	.929	.714-1.214	0.0	2.2
	Staff Competence	273	2.401	0.587	2.500	2.000-3.000	0.0	32.6
	Information and Education	273	1.329	0.515	1.333	.944-1.750	0.0	0.0
	Emotional and Psychological Support	273	2.037	0.513	2.143	1.667-2.500	0.0	0.7
	Continuity and Coordination	273	2.285	0.584	2.375	1.875-2.750	0.0	12.5
	Participation in Care	273	1.647	0.435	1.667	1.333-2.000	0.0	0.0
Staff	Accessibility	74	2.197	0.397	2.188	1.938-2.563	0.0	1.4
	Minimizing Cost	74	2.291	0.832	3.000	1.500-3.000	2.7	55.4
	Physical Comfort proxy	53	1.925	1.174	2.000	1.000-3.000	18.9	45.3
	Privacy	70	1.538	0.496	1.667	1.333-1.667	0.0	4.3
	Staff Attitude and Communication	74	2.150	0.258	2.167	2.039-2.286	0.0	1.4
	Staff Competence	74	2.385	0.457	2.500	2.000-2.750	0.0	12.2
	Information and Education	74	2.310	0.500	2.389	2.000-2.667	0.0	0.0
	Emotional and Psychological Support	74	1.809	0.455	1.833	1.579-2.000	0.0	0.0
	Continuity and Coordination	74	1.457	0.427	1.406	1.188-1.676	0.0	0.0
	Participation in Care	74	2.225	0.524	2.333	2.000-2.667	0.0	6.8

Table S3. Data-quality, scoring, and outcome-distribution audit.

Finding	Result	Analytical handling
Patient records	273	All available records retained in the primary analysis
Unique exact patient patterns	173	One-pattern-per-record sensitivity analyses performed
Records beyond first pattern occurrence	100	Not classified as confirmed duplicate participants
Location of excess exact-pattern occurrences	IRM only	Persona 145/145 unique; IRM 127 records comprised 27 unique patterns
Identifiers/timestamps for record linkage	Unavailable	Record-level source verification was not possible
Patient global rating	Mean 9.59; SD 1.14	Ceiling acknowledged; exploratory models only
Patients rating care 10/10	79.1% (216/273)	Top-box sensitivity analysis; 46 ratings below 10 in model sample
Staff global ratings outside stated 0-10 range	50/74 coded 11	Staff global rating excluded pending source verification
Unexpected item responses outside implemented mappings	308 occurrences	68 experience responses (38 patient and 30 staff) plus 240 patient importance responses; all were set to missing; see Table S12
Administered item-level scoring codebook	Unavailable	All harmonized-score analyses conditional on Supplementary Code S1

Table S4. Paired patient importance-experience differences.

Rank	Domain	Experience item	Paired n	Experience mean	Importance mean	Mean paired difference	95% CI	t (df)	Raw p	FDR p
1	Privacy	Q115	215	0.595	2.274	1.679	1.479 to 1.879	16.563 (214)	<0.001	<.001
2	Minimizing Cost	Q107	271	1.052	2.395	1.343	1.195 to 1.491	17.844 (270)	<0.001	<.001
3	Information and Education	Q36	272	0.728	2.059	1.331	1.174 to 1.488	16.654 (271)	<0.001	<.001
4	Participation in Care	Q55	272	1.257	2.438	1.180	1.068 to 1.293	20.675 (271)	<0.001	<.001
5	Information and Education	Q30	241	1.158	2.083	0.925	0.742 to 1.109	9.946 (240)	<0.001	<.001
6	Information and Education	Q32	271	0.908	1.598	0.690	0.525 to 0.855	8.249 (270)	<0.001	<.001
7	Emotional and Psychological Support	Q63	273	1.425	2.073	0.648	0.510 to 0.786	9.257 (272)	<0.001	<.001
8	Accessibility	Q103	268	1.394	1.970	0.576	0.421 to 0.732	7.280 (267)	<0.001	<.001
9	Physical Comfort proxy	Q99	273	1.531	2.037	0.505	0.322 to 0.689	5.419 (272)	<0.001	<.001
10	Information and Education	Q26	273	0.883	1.341	0.458	0.346 to 0.570	8.058 (272)	<0.001	<.001
11	Emotional and Psychological Support	Q73	210	1.948	2.224	0.276	0.095 to 0.458	3.000 (209)	0.003	.005

12	Staff Competence	Q93	272	2.188	2.438	0.250	0.109 to 0.391	3.486 (271)	<0.001	<.001
13	Emotional and Psychological Support	Q61	122	1.906	2.148	0.242	-0.029 to 0.513	1.768 (121)	0.080	.097
14	Accessibility	Q105	225	2.049	2.253	0.204	0.020 to 0.389	2.187 (224)	0.030	.041
15	Staff Attitude and Communication	Q44	264	1.102	1.288	0.186	0.061 to 0.310	2.939 (263)	0.004	.005
16	Continuity and Coordination	Q75	273	2.209	2.297	0.088	-0.077 to 0.253	1.049 (272)	0.295	.332
17	Continuity and Coordination	Q97	273	2.282	2.315	0.033	-0.109 to 0.175	0.458 (272)	0.647	.662
18	Emotional and Psychological Support	Q65	272	2.062	2.085	0.022	-0.125 to 0.170	0.294 (271)	0.769	.769
19	Privacy	Q113	213	1.920	1.859	-0.061	-0.234 to 0.112	-0.695 (212)	0.488	.510
20	Emotional and Psychological Support	Q71	271	2.085	2.011	-0.074	-0.212 to 0.065	-1.048 (270)	0.295	.332
21	Continuity and Coordination	Q85	271	2.100	2.015	-0.085	-0.269 to 0.099	-0.907 (270)	0.365	.391
22	Information and Education	Q21	267	2.124	2.037	-0.086	-0.265 to 0.093	-0.946 (266)	0.345	.379
23	Continuity and Coordination	Q79	273	2.242	2.147	-0.095	-0.265 to 0.074	-1.106 (272)	0.270	.320
24	Information and Education	Q28	272	0.735	0.614	-0.121	-0.228 to -0.015	-2.246 (271)	0.025	.036

25	Continuity and Coordination	Q81	273	2.462	2.330	-0.132	-0.275 to 0.011	-1.817 (272)	0.070	.088
26	Staff Competence	Q95	273	2.392	2.227	-0.165	-0.317 to -0.013	-2.138 (272)	0.033	.044
27	Emotional and Psychological Support	Q67	273	2.304	2.136	-0.168	-0.299 to -0.038	-2.542 (272)	0.012	.017
28	Staff Attitude and Communication	Q50	271	0.697	0.513	-0.185	-0.305 to -0.064	-3.022 (270)	0.003	.004
29	Continuity and Coordination	Q83	271	1.970	1.768	-0.203	-0.405 to -0.001	-1.979 (270)	0.049	.063
30	Accessibility	Q101	271	2.480	2.251	-0.229	-0.366 to -0.091	-3.281 (270)	0.001	.002
31	Staff Attitude and Communication	Q48	272	0.577	0.316	-0.261	-0.362 to -0.160	-5.082 (271)	<0.001	<.001
32	Emotional and Psychological Support	Q69	267	2.423	2.139	-0.285	-0.441 to -0.128	-3.582 (266)	<0.001	<.001
33	Staff Competence	Q91	273	2.582	2.278	-0.304	-0.438 to -0.170	-4.468 (272)	<0.001	<.001
34	Accessibility	Q17	273	2.161	1.817	-0.344	-0.480 to -0.208	-4.989 (272)	<0.001	<.001
35	Staff Attitude and Communication	Q42	272	1.224	0.875	-0.349	-0.522 to -0.176	-3.979 (271)	<0.001	<.001
36	Information and Education	Q34	272	0.798	0.393	-0.404	-0.530 to -0.279	-6.344 (271)	<0.001	<.001
37	Staff Competence	Q89	273	2.447	2.018	-0.429	-0.593 to -0.264	-5.137 (272)	<0.001	<.001

38	Participation in Care	Q57	272	2.526	2.085	-0.441	-0.564 to -0.319	-7.094 (271)	<0.001	<.001
39	Staff Attitude and Communication	Q46	269	0.721	0.249	-0.472	-0.584 to -0.361	-8.340 (268)	<0.001	<.001
40	Accessibility	Q19	259	2.647	2.104	-0.542	-0.675 to -0.410	-8.036 (258)	<0.001	<.001
41	Privacy	Q111	271	2.506	1.956	-0.550	-0.709 to -0.390	-6.781 (270)	<0.001	<.001
42	Continuity and Coordination	Q87	273	2.476	1.923	-0.553	-0.727 to -0.379	-6.272 (272)	<0.001	<.001
43	Information and Education	Q23	272	2.493	1.901	-0.592	-0.734 to -0.449	-8.183 (271)	<0.001	<.001
44	Continuity and Coordination	Q77	271	2.552	1.745	-0.806	-0.956 to -0.657	-10.609 (270)	<0.001	<.001
45	Privacy	Q109	250	2.420	1.232	-1.188	-1.394 to -0.982	-11.349 (249)	<0.001	<.001

Footnote: Each difference was calculated within patients with both experience and importance values for that item. Positive values indicate that importance exceeded the favorability of experience. The paired t statistic is reported with degrees of freedom in parentheses. Both unadjusted p values and Benjamini-Hochberg FDR-adjusted p values are shown.

Table S5. One-pattern-per-record sensitivity analysis of patient-staff differences.

Domain	Staff mean	Patient mean	Difference	95% CI	Hedges g	FDR p
Accessibility	2.197	2.143	0.054	-0.068 to 0.176	0.109	0.426
Minimizing Cost	2.291	1.101	1.189	0.953 to 1.426	1.317	<0.001
Physical Comfort proxy	1.925	1.607	0.318	-0.044 to 0.679	0.284	0.105
Privacy	1.538	1.969	-0.431	-0.584 to -0.278	-0.725	<0.001
Staff Attitude and Communication	2.150	0.953	1.196	1.109 to 1.284	3.083	<0.001
Staff Competence	2.385	2.399	-0.014	-0.150 to 0.123	-0.025	0.843
Information and Education	2.310	1.229	1.081	0.942 to 1.220	2.102	<0.001
Emotional and Psychological Support	1.809	1.992	-0.184	-0.315 to -0.052	-0.359	0.009
Continuity and Coordination	1.457	2.367	-0.910	-1.035 to -0.785	-1.847	<0.001
Participation in Care	2.225	1.683	0.542	0.404 to 0.680	1.150	<0.001

Table S6. Exact-pattern sensitivity analysis of the global-rating regression.

Predictor	B	HC3 SE	95% CI	p
Age	0.071	0.134	-0.193 to 0.335	0.598
Higher professional/university vs secondary/vocational	0.036	0.253	-0.463 to 0.536	0.886
Postgraduate/other vs secondary/vocational	0.071	0.286	-0.495 to 0.636	0.805
Persona vs IRM	-0.081	0.254	-0.582 to 0.420	0.751
Accessibility	0.176	0.228	-0.275 to 0.627	0.441
Minimizing Cost	-0.146	0.102	-0.348 to 0.056	0.156
Physical Comfort proxy	0.138	0.115	-0.089 to 0.365	0.233
Privacy	0.273	0.244	-0.209 to 0.755	0.265
Staff Attitude and Communication	-0.102	0.394	-0.881 to 0.677	0.797
Staff Competence	0.021	0.218	-0.409 to 0.452	0.922
Information and Education	-0.090	0.196	-0.478 to 0.299	0.649
Emotional and Psychological Support	0.368	0.184	0.005 to 0.732	0.047
Continuity and Coordination	-0.047	0.186	-0.414 to 0.321	0.802
Participation in Care	0.173	0.238	-0.298 to 0.643	0.470

Table S7. Top-box logistic sensitivity analysis with HC3 robust inference.

Predictor	OR	95% CI	HC3 p
age_ord	1.71	0.99-2.97	0.055
education_collapsedHigher_professional_or_university	0.79	0.25-2.43	0.675
education_collapsedPostgraduate_or_other	0.15	0.03-0.71	0.017
Persona vs IRM	1.75	0.61-5.04	0.300
domain_Accessibility	2.10	0.67-6.56	0.202
domain_Minimizing_cost	0.69	0.43-1.10	0.119
domain_Physical_comfort_proxy	1.72	1.12-2.65	0.014
domain_Privacy	0.62	0.30-1.27	0.189
domain_Staff_attitude_communication	2.71	0.96-7.63	0.059
domain_Staff_competence	0.65	0.23-1.86	0.422
domain_Information_education	1.48	0.56-3.89	0.427
domain_Emotional_psychological_support	2.02	0.79-5.15	0.142
domain_Continuity_coordination	1.92	0.75-4.92	0.177
domain_Participation_in_care	0.42	0.16-1.08	0.072

Footnote: OR > 1 indicates greater odds of a 10/10 rating. The model included 207 top-box and 46 below-top-box observations with 14 non-intercept predictor parameters and is exploratory.

Table S8. Clinic-stratified patient-staff comparisons in submitted-record and one-pattern-per-record analyses.

Scenario	Clinic	Domain	Patient n; mean (SD)	Staff n; mean (SD)	Staff-patient difference (95% CI)	Hedges g	FDR p
Submitted records	Persona	Accessibility	141; 2.132 (0.538)	43; 2.263 (0.359)	0.131 (-0.010 to 0.272)	0.260	0.076
Submitted records	Persona	Minimizing Cost	145; 1.128 (0.944)	43; 2.233 (0.826)	1.105 (0.809 to 1.400)	1.198	<0.001
Submitted records	Persona	Physical Comfort proxy	145; 1.628 (1.080)	26; 2.462 (0.948)	0.834 (0.416 to 1.252)	0.782	<0.001
Submitted records	Persona	Privacy	132; 1.978 (0.636)	42; 1.548 (0.494)	-0.430 (-0.618 to -0.243)	-0.708	<0.001
Submitted records	Persona	Staff Attitude and Communication	145; 0.920 (0.406)	43; 2.137 (0.286)	1.217 (1.108 to 1.327)	3.168	<0.001
Submitted records	Persona	Staff Competence	145; 2.390 (0.577)	43; 2.436 (0.466)	0.046 (-0.124 to 0.217)	0.083	0.590
Submitted records	Persona	Information and Education	145; 1.169 (0.505)	43; 2.251 (0.569)	1.082 (0.889 to 1.275)	2.071	<0.001
Submitted records	Persona	Emotional and Psychological Support	145; 1.971 (0.536)	43; 1.714 (0.407)	-0.257 (-0.409 to -0.106)	-0.503	0.001
Submitted records	Persona	Continuity and Coordination	145; 2.400 (0.488)	43; 1.316 (0.341)	-1.084 (-1.214 to -0.953)	-2.353	<0.001
Submitted records	Persona	Participation in Care	145; 1.700 (0.449)	43; 2.031 (0.553)	0.331 (0.146 to 0.516)	0.694	<0.001
Submitted records	IRM	Accessibility	127; 2.159 (0.489)	31; 2.105 (0.434)	-0.054 (-0.233 to 0.125)	-0.113	0.606
Submitted records	IRM	Minimizing Cost	127; 0.957 (0.838)	31; 2.371 (0.846)	1.414 (1.074 to 1.755)	1.676	<0.001
Submitted records	IRM	Physical Comfort proxy	127; 1.425 (1.205)	27; 1.407 (1.152)	-0.018 (-0.516 to 0.480)	-0.015	0.943
Submitted records	IRM	Privacy	121; 1.891 (0.618)	28; 1.524 (0.508)	-0.367 (-0.591 to -0.144)	-0.610	0.003
Submitted records	IRM	Staff Attitude and Communication	127; 1.133 (0.544)	31; 2.167 (0.217)	1.035 (0.912 to 1.157)	2.068	<0.001

Submitted records	IRM	Staff Competence	127; 2.415 (0.601)	31; 2.315 (0.442)	-0.101 (-0.292 to 0.091)	-0.175	0.370
Submitted records	IRM	Information and Education	127; 1.504 (0.460)	31; 2.392 (0.377)	0.888 (0.730 to 1.047)	1.987	<0.001
Submitted records	IRM	Emotional and Psychological Support	127; 2.116 (0.476)	31; 1.941 (0.492)	-0.176 (-0.373 to 0.022)	-0.365	0.114
Submitted records	IRM	Continuity and Coordination	127; 2.153 (0.656)	31; 1.652 (0.462)	-0.501 (-0.703 to -0.298)	-0.799	<0.001
Submitted records	IRM	Participation in Care	127; 1.583 (0.411)	31; 2.495 (0.332)	0.912 (0.772 to 1.052)	2.284	<0.001
One pattern per exact response	Persona	Accessibility	141; 2.132 (0.538)	43; 2.263 (0.359)	0.131 (-0.010 to 0.272)	0.260	0.076
One pattern per exact response	Persona	Minimizing Cost	145; 1.128 (0.944)	43; 2.233 (0.826)	1.105 (0.809 to 1.400)	1.198	<0.001
One pattern per exact response	Persona	Physical Comfort proxy	145; 1.628 (1.080)	26; 2.462 (0.948)	0.834 (0.416 to 1.252)	0.782	<0.001
One pattern per exact response	Persona	Privacy	132; 1.978 (0.636)	42; 1.548 (0.494)	-0.430 (-0.618 to -0.243)	-0.708	<0.001
One pattern per exact response	Persona	Staff Attitude and Communication	145; 0.920 (0.406)	43; 2.137 (0.286)	1.217 (1.108 to 1.327)	3.168	<0.001
One pattern per exact response	Persona	Staff Competence	145; 2.390 (0.577)	43; 2.436 (0.466)	0.046 (-0.124 to 0.217)	0.083	0.590
One pattern per exact response	Persona	Information and Education	145; 1.169 (0.505)	43; 2.251 (0.569)	1.082 (0.889 to 1.275)	2.071	<0.001
One pattern per exact response	Persona	Emotional and Psychological Support	145; 1.971 (0.536)	43; 1.714 (0.407)	-0.257 (-0.409 to -0.106)	-0.503	0.001
One pattern per exact response	Persona	Continuity and Coordination	145; 2.400 (0.488)	43; 1.316 (0.341)	-1.084 (-1.214 to -0.953)	-2.353	<0.001
One pattern per exact response	Persona	Participation in Care	145; 1.700 (0.449)	43; 2.031 (0.553)	0.331 (0.146 to 0.516)	0.694	<0.001
One pattern per exact response	IRM	Accessibility	27; 2.177 (0.499)	31; 2.105 (0.434)	-0.073 (-0.321 to 0.175)	-0.154	0.621
One pattern per exact response	IRM	Minimizing Cost	27; 0.944 (0.847)	31; 2.371 (0.846)	1.427 (0.980 to 1.873)	1.662	<0.001
One pattern per exact response	IRM	Physical Comfort proxy	27; 1.519 (1.221)	27; 1.407 (1.152)	-0.111 (-0.759 to 0.537)	-0.092	0.732

One pattern per exact response	IRM	Privacy	26; 1.923 (0.619)	28; 1.524 (0.508)	-0.399 (-0.710 to -0.088)	-0.697	0.022
One pattern per exact response	IRM	Staff Attitude and Communication	27; 1.123 (0.519)	31; 2.167 (0.217)	1.044 (0.826 to 1.262)	2.658	<0.001
One pattern per exact response	IRM	Staff Competence	27; 2.454 (0.605)	31; 2.315 (0.442)	-0.139 (-0.423 to 0.144)	-0.262	0.410
One pattern per exact response	IRM	Information and Education	27; 1.513 (0.458)	31; 2.392 (0.377)	0.880 (0.657 to 1.103)	2.084	<0.001
One pattern per exact response	IRM	Emotional and Psychological Support	27; 2.122 (0.498)	31; 1.941 (0.492)	-0.182 (-0.443 to 0.080)	-0.362	0.242
One pattern per exact response	IRM	Continuity and Coordination	27; 2.183 (0.633)	31; 1.652 (0.462)	-0.531 (-0.828 to -0.234)	-0.955	0.002
One pattern per exact response	IRM	Participation in Care	27; 1.580 (0.420)	31; 2.495 (0.332)	0.914 (0.713 to 1.116)	2.405	<0.001

Footnote: Differences are staff minus patient. P values are FDR adjusted within each scenario and clinic family.

Table S9. Item-level patient-staff comparisons in submitted-record and one-pattern-per-record analyses.

Scenario	Domain	Patient/staff item	Item wording	Patient n; mean	Staff n; mean	Difference (95% CI)	Hedges g	FDR p
Submitted	Accessibility	Q19/S2	Was it a problem for you to contact the staff (by phone or email, via WhatsApp) if you had any questions?	259; 2.647	69; 2.283	-0.364 (-0.581 to -0.147)	-0.509	0.002
Submitted	Accessibility	Q101/S44	How often have you had to wait more than 3 weeks to get an appointment with a doctor?	273; 2.476	74; 2.149	-0.328 (-0.459 to -0.196)	-0.446	<0.001
Submitted	Accessibility	Q103/S45	How much time passed between your first hospital visit and when you received your treatment plan?	273; 1.376	74; 1.895	0.519 (0.309 to 0.729)	0.564	<0.001
Submitted	Accessibility	Q105/S46	How long on average did you have to wait ‘unnecessarily’ before being able to start a second treatment?	226; 2.044	68; 2.485	0.441 (0.171 to 0.711)	0.392	0.002
Submitted	Minimizing Cost	Q107/S47	How would you rate the cost of infertility treatment at this medical facility?	273; 1.049	74; 2.291	1.241 (1.022 to 1.461)	1.401	<0.001
Submitted	Physical Comfort proxy	Q99/S43	How long did you usually have to wait in the waiting room/reception area?	273; 1.531	53; 1.925	0.393 (0.044 to 0.743)	0.342	0.034
Submitted	Privacy	Q109/S48	Will this medical facility provide a female doctor for examination if you request it?	250; 2.420	27; 1.407	-1.013 (-1.467 to -0.559)	-1.131	<0.001
Submitted	Privacy	Q111/S49	To what extent do you feel your privacy life was respected?	272; 2.507	68; 2.809	0.301 (0.138 to 0.465)	0.367	<0.001
Submitted	Privacy	Q113/S50	Did your doctor or nurse ask your permission before allowing others to enter the room or procedure room?	213; 1.920	70; 1.657	-0.263 (-0.494 to -0.032)	-0.287	0.032
Submitted	Privacy	Q115/S51	Has your doctor or nurse talked to others about your infertility problems?	215; 0.595	67; 0.239	-0.357 (-0.580 to -0.133)	-0.351	0.003
Submitted	Staff Attitude and Communication	Q42/S14	Were carers honest and clear about what to expect from the fertility service?	272; 1.224	74; 2.784	1.560 (1.401 to 1.718)	1.619	<0.001
Submitted	Staff Attitude and Communication	Q44/S15	Have the research results been discussed with you?	264; 1.102	74; 2.959	1.857 (1.718 to 1.996)	1.976	<0.001
Submitted	Staff Attitude and Communication	Q46/S16	How often did the staff listen carefully?	273; 0.725	74; 2.668	1.942 (1.797 to 2.088)	2.486	<0.001

Submitted	Staff Attitude and Communication	Q48/S17	How often did the staff take you seriously?	272; 0.577	74; 2.430	1.853 (1.696 to 2.009)	2.757	<0.001
Submitted	Staff Attitude and Communication	Q50/S18	How often did you feel that the staff had enough time for you?	271; 0.697	74; 1.362	0.665 (0.525 to 0.804)	0.940	<0.001
Submitted	Staff Attitude and Communication	Q52/S19	How often have you felt like staff were talking "about you" instead of talking to you?	272; 2.357	73; 0.315	-2.042 (-2.224 to -1.859)	-2.765	<0.001
Submitted	Staff Attitude and Communication	Q53/S20	If something went wrong during your treatment, were staff willing to talk to you about mistakes or incidents?	246; 0.366	36; 3.000	2.634 (2.511 to 2.758)	2.855	<0.001
Submitted	Staff Competence	Q89/S38	How often did employees use difficult words without explaining them to you?	273; 2.447	74; 2.351	-0.096 (-0.314 to 0.122)	-0.106	0.423
Submitted	Staff Competence	Q91/S39	How often was the doctor available for the appointments?	273; 2.582	74; 2.676	0.093 (-0.084 to 0.271)	0.112	0.335
Submitted	Staff Competence	Q93/S40	Did you find the doctor(s) competent?	273; 2.183	74; 2.149	-0.035 (-0.307 to 0.238)	-0.030	0.820
Submitted	Staff Competence	Q95/S41	How often did the staff seem disorganized?	273; 2.392	74; 2.365	-0.027 (-0.226 to 0.172)	-0.030	0.820
Submitted	Information and Education	Q21/S3	Have you received contact numbers for urgent questions or problems at night or on weekends?	267; 2.124	74; 2.108	-0.015 (-0.374 to 0.343)	-0.011	0.932
Submitted	Information and Education	Q23/S4	Did you also receive written information in addition to verbal information?	273; 2.489	74; 2.696	0.207 (-0.009 to 0.423)	0.215	0.071
Submitted	Information and Education	Q25/S5	Was there any information about the research?	273; 2.037	74; 2.459	0.423 (0.226 to 0.620)	0.452	<0.001
Submitted	Information and Education	Q26/S6	Have various treatment options been discussed with you?	273; 0.883	74; 1.514	0.631 (0.396 to 0.866)	0.787	<0.001
Submitted	Information and Education	Q28/S7	Was the information about the treatment you would receive complete and comprehensive?	273; 0.740	74; 2.405	1.665 (1.469 to 1.862)	2.070	<0.001
Submitted	Information and Education	Q30/S8	Have you received a treatment plan review?	242; 1.165	74; 2.676	1.510 (1.226 to 1.795)	1.107	<0.001

Submitted	Information and Education	Q32/S9	Have you been informed about any possible side effects of the medicine prescribed to you?	272; 0.915	64; 2.109	1.194 (0.863 to 1.525)	1.047	<0.001
Submitted	Information and Education	Q34/S10	Were the instructions on how to administer hormones or medications complete and comprehensive?	272; 0.798	69; 2.725	1.927 (1.754 to 2.099)	2.433	<0.001
Submitted	Information and Education	Q36/S11	Did the staff tell you how to get support from a social worker or psychologist?	272; 0.728	74; 2.189	1.461 (1.153 to 1.769)	1.461	<0.001
Submitted	Emotional and Psychological Support	Q61/S24	Did you have access to your own medical records during the treatment period?	122; 1.906	33; 2.727	0.822 (0.470 to 1.173)	0.715	<0.001
Submitted	Emotional and Psychological Support	Q63/S25	How often has your doctor shown interest in your personal situation? (e.g. the combination of your treatment, home and/or work circumstances)	273; 1.425	72; 2.347	0.922 (0.671 to 1.174)	1.173	<0.001
Submitted	Emotional and Psychological Support	Q65/S26	How often has your doctor shown empathy for your emotions and your current situation?	273; 2.059	74; 2.270	0.212 (-0.022 to 0.446)	0.204	0.087
Submitted	Emotional and Psychological Support	Q67/S27	Were the nurses understanding of your situation?	273; 2.304	68; 2.735	0.431 (0.243 to 0.620)	0.492	<0.001
Submitted	Emotional and Psychological Support	Q69/S28	Did the staff also involve your partner/husband in the treatment and examination?	267; 2.423	74; 1.365	-1.058 (-1.301 to -0.815)	-1.170	<0.001
Submitted	Emotional and Psychological Support	Q71/S29	How often did you receive any personal attention and support from nurses during your treatment?	273; 2.073	72; 1.417	-0.657 (-0.938 to -0.375)	-0.632	<0.001
Submitted	Emotional and Psychological Support	Q73/S30	Did staff pay attention to any possible emotional consequences of fertility issues?	210; 1.948	74; 0.486	-1.461 (-1.660 to -1.263)	-1.379	<0.001
Submitted	Continuity and Coordination	Q75/S31	Was there one person assigned to you to contact you at any time when you had any questions or problems (e.g. with a nurse)?	273; 2.209	63; 1.476	-0.733 (-1.143 to -0.322)	-0.537	<.001
Submitted	Continuity and Coordination	Q77/S32	How many different doctors were involved in your treatment?	273; 2.544	74; 1.277	-1.267 (-1.525 to -1.009)	-1.357	<0.001
Submitted	Continuity and Coordination	Q79/S33	Did you have one lead doctor?	273; 2.242	64; 1.219	-1.023 (-1.359 to -0.687)	-0.844	<0.001

Submitted	Continuity and Coordination	Q81/S34	Did you only go to the same doctor at every appointment?	273; 2.462	73; 1.096	-1.366 (-1.681 to -1.051)	-1.317	<0.001
Submitted	Continuity and Coordination	Q83/S35	How often have you had to repeat the same story to different doctors?.	273; 1.971	74; 2.284	0.313 (0.084 to 0.542)	0.302	0.010
Submitted	Continuity and Coordination	Q85/S36	How often have you received conflicting information or advice?	273; 2.099	74; 0.568	-1.531 (-1.771 to -1.292)	-1.418	<0.001
Submitted	Continuity and Coordination	Q87/S37	Did employees contradict each other on policy (one says one thing, another says something else)?	273; 2.476	74; 0.919	-1.557 (-1.852 to -1.263)	-1.505	<0.001
Submitted	Continuity and Coordination	Q97/S42	How often was your treatment well coordinated in the clinic?	273; 2.282	74; 2.743	0.461 (0.285 to 0.637)	0.486	<0.001
Submitted	Participation in Care	Q54/S21	How often was your doctor open to your opinions and ideas about treatment?	272; 1.162	74; 1.851	0.690 (0.446 to 0.933)	0.694	<0.001
Submitted	Participation in Care	Q55/S22	How often were you given the opportunity to ask questions to your doctor?	273; 1.256	74; 2.257	1.000 (0.792 to 1.209)	1.493	<0.001
Submitted	Participation in Care	Q57/S23	Were you involved in decision making if you preferred?	273; 2.520	74; 2.568	0.047 (-0.126 to 0.221)	0.064	0.629
One pattern	Accessibility	Q19/S2	Was it a problem for you to contact the staff (by phone or email, via WhatsApp) if you had any questions?	163; 2.623	69; 2.283	-0.340 (-0.569 to -0.112)	-0.450	0.005
One pattern	Accessibility	Q101/S44	How often have you had to wait more than 3 weeks to get an appointment with a doctor?	173; 2.543	74; 2.149	-0.395 (-0.540 to -0.250)	-0.586	<0.001
One pattern	Accessibility	Q103/S45	How much time passed between your first hospital visit and when you received your treatment plan?	173; 1.374	74; 1.895	0.521 (0.293 to 0.749)	0.571	<0.001
One pattern	Accessibility	Q105/S46	How long on average did you have to wait 'unnecessarily' before being able to start a second treatment?	134; 1.970	68; 2.485	0.515 (0.215 to 0.815)	0.464	0.001
One pattern	Minimizing Cost	Q107/S47	How would you rate the cost of infertility treatment at this medical facility?	173; 1.101	74; 2.291	1.189 (0.953 to 1.426)	1.317	<0.001
One pattern	Physical Comfort proxy	Q99/S43	How long did you usually have to wait in the waiting room/reception area?	173; 1.607	53; 1.925	0.318 (-0.044 to 0.679)	0.284	0.096

One pattern	Privacy	Q109/S48	Will this medical facility provide a female doctor for examination if you request it?	156; 2.487	27; 1.407	-1.080 (-1.539 to -0.620)	-1.225	<0.001
One pattern	Privacy	Q111/S49	To what extent do you feel your privacy life was respected?	172; 2.581	68; 2.809	0.227 (0.052 to 0.402)	0.306	0.014
One pattern	Privacy	Q113/S50	Did your doctor or nurse ask your permission before allowing others to enter the room or procedure room?	141; 1.894	70; 1.657	-0.236 (-0.479 to 0.006)	-0.272	0.068
One pattern	Privacy	Q115/S51	Has your doctor or nurse talked to others about your infertility problems?	141; 0.610	67; 0.239	-0.371 (-0.622 to -0.120)	-0.368	0.005
One pattern	Staff Attitude and Communication	Q42/S14	Were carers honest and clear about what to expect from the fertility service?	172; 1.006	74; 2.784	1.778 (1.606 to 1.950)	2.126	<0.001
One pattern	Staff Attitude and Communication	Q44/S15	Have the research results been discussed with you?	167; 0.961	74; 2.959	1.998 (1.833 to 2.164)	2.319	<0.001
One pattern	Staff Attitude and Communication	Q46/S16	How often did the staff listen carefully?	173; 0.636	74; 2.668	2.032 (1.879 to 2.184)	3.033	<0.001
One pattern	Staff Attitude and Communication	Q48/S17	How often did the staff take you seriously?	172; 0.552	74; 2.430	1.877 (1.711 to 2.044)	2.913	<0.001
One pattern	Staff Attitude and Communication	Q50/S18	How often did you feel that the staff had enough time for you?	171; 0.690	74; 1.362	0.672 (0.516 to 0.828)	0.978	<0.001
One pattern	Staff Attitude and Communication	Q52/S19	How often have you felt like staff were talking "about you" instead of talking to you?	172; 2.337	73; 0.315	-2.022 (-2.217 to -1.827)	-2.733	<0.001
One pattern	Staff Attitude and Communication	Q53/S20	If something went wrong during your treatment, were staff willing to talk to you about mistakes or incidents?	149; 0.362	36; 3.000	2.638 (2.479 to 2.796)	2.977	<0.001
One pattern	Staff Competence	Q89/S38	How often did employees use difficult words without explaining them to you?	173; 2.462	74; 2.351	-0.111 (-0.340 to 0.118)	-0.130	0.368
One pattern	Staff Competence	Q91/S39	How often was the doctor available for the appointments?	173; 2.590	74; 2.676	0.086 (-0.107 to 0.279)	0.107	0.397
One pattern	Staff Competence	Q93/S40	Did you find the doctor(s) competent?	173; 2.046	74; 2.149	0.102 (-0.200 to 0.404)	0.085	0.515
One pattern	Staff Competence	Q95/S41	How often did the staff seem disorganized?	173; 2.497	74; 2.365	-0.132 (-0.344 to 0.079)	-0.156	0.244

One pattern	Information and Education	Q21/S3	Have you received contact numbers for urgent questions or problems at night or on weekends?	170; 1.924	74; 2.108	0.185 (-0.201 to 0.570)	0.129	0.368
One pattern	Information and Education	Q23/S4	Did you also receive written information in addition to verbal information?	173; 2.471	74; 2.696	0.225 (-0.014 to 0.463)	0.230	0.076
One pattern	Information and Education	Q25/S5	Was there any information about the research?	173; 1.798	74; 2.459	0.662 (0.439 to 0.884)	0.690	<0.001
One pattern	Information and Education	Q26/S6	Have various treatment options been discussed with you?	173; 0.815	74; 1.514	0.698 (0.452 to 0.945)	0.839	<0.001
One pattern	Information and Education	Q28/S7	Was the information about the treatment you would receive complete and comprehensive?	173; 0.659	74; 2.405	1.746 (1.544 to 1.949)	2.366	<0.001
One pattern	Information and Education	Q30/S8	Have you received a treatment plan review?	157; 1.108	74; 2.676	1.567 (1.254 to 1.881)	1.192	<0.001
One pattern	Information and Education	Q32/S9	Have you been informed about any possible side effects of the medicine prescribed to you?	172; 0.855	64; 2.109	1.255 (0.914 to 1.595)	1.147	<0.001
One pattern	Information and Education	Q34/S10	Were the instructions on how to administer hormones or medications complete and comprehensive?	172; 0.640	69; 2.725	2.085 (1.904 to 2.267)	2.892	<0.001
One pattern	Information and Education	Q36/S11	Did the staff tell you how to get support from a social worker or psychologist?	172; 0.724	74; 2.189	1.465 (1.144 to 1.786)	1.393	<0.001
One pattern	Emotional and Psychological Support	Q61/S24	Did you have access to your own medical records during the treatment period?	75; 1.620	33; 2.727	1.107 (0.715 to 1.500)	0.987	<0.001
One pattern	Emotional and Psychological Support	Q63/S25	How often has your doctor shown interest in your personal situation? (e.g. the combination of your treatment, home and/or work circumstances)	173; 1.370	72; 2.347	0.977 (0.719 to 1.236)	1.213	<0.001
One pattern	Emotional and Psychological Support	Q65/S26	How often has your doctor shown empathy for your emotions and your current situation?	173; 1.908	74; 2.270	0.363 (0.098 to 0.627)	0.328	0.010
One pattern	Emotional and Psychological Support	Q67/S27	Were the nurses understanding of your situation?	173; 2.231	68; 2.735	0.504 (0.293 to 0.715)	0.564	<0.001
One pattern	Emotional and Psychological Support	Q69/S28	Did the staff also involve your partner/husband in the treatment and examination?	172; 2.453	74; 1.365	-1.089 (-1.346 to -0.831)	-1.176	<0.001

One pattern	Emotional and Psychological Support	Q71/S29	How often did you receive any personal attention and support from nurses during your treatment?	173; 2.116	72; 1.417	-0.699 (-0.998 to -0.400)	-0.655	<0.001
One pattern	Emotional and Psychological Support	Q73/S30	Did staff pay attention to any possible emotional consequences of fertility issues?	136; 1.993	74; 0.486	-1.506 (-1.734 to -1.278)	-1.525	<0.001
One pattern	Continuity and Coordination	Q75/S31	Was there one person assigned to you to contact you at any time when you had any questions or problems (e.g. with a nurse)?	173; 2.220	63; 1.476	-0.743 (-1.171 to -0.316)	-0.540	0.001
One pattern	Continuity and Coordination	Q77/S32	How many different doctors were involved in your treatment?	173; 2.645	74; 1.277	-1.367 (-1.629 to -1.106)	-1.584	<0.001
One pattern	Continuity and Coordination	Q79/S33	Did you have one lead doctor?	173; 2.506	64; 1.219	-1.287 (-1.629 to -0.945)	-1.168	<0.001
One pattern	Continuity and Coordination	Q81/S34	Did you only go to the same doctor at every appointment?	173; 2.630	73; 1.096	-1.534 (-1.852 to -1.217)	-1.580	<0.001
One pattern	Continuity and Coordination	Q83/S35	How often have you had to repeat the same story to different doctors?.	173; 1.954	74; 2.284	0.330 (0.078 to 0.582)	0.318	0.013
One pattern	Continuity and Coordination	Q85/S36	How often have you received conflicting information or advice?	173; 2.104	74; 0.568	-1.536 (-1.796 to -1.276)	-1.453	<0.001
One pattern	Continuity and Coordination	Q87/S37	Did employees contradict each other on policy (one says one thing, another says something else)?	173; 2.503	74; 0.919	-1.584 (-1.892 to -1.276)	-1.502	<0.001
One pattern	Continuity and Coordination	Q97/S42	How often was your treatment well coordinated in the clinic?	173; 2.376	74; 2.743	0.368 (0.174 to 0.561)	0.420	<0.001
One pattern	Participation in Care	Q54/S21	How often was your doctor open to your opinions and ideas about treatment?	172; 1.221	74; 1.851	0.630 (0.363 to 0.897)	0.603	<0.001
One pattern	Participation in Care	Q55/S22	How often were you given the opportunity to ask questions to your doctor?	173; 1.237	74; 2.257	1.020 (0.805 to 1.235)	1.481	<0.001
One pattern	Participation in Care	Q57/S23	Were you involved in decision making if you preferred?	173; 2.590	74; 2.568	-0.022 (-0.204 to 0.160)	-0.032	0.811

Footnote: Items were matched only when patient and staff wording addressed the same service feature and could be oriented in the same favorable-care direction. FDR adjustment was applied across all item comparisons within each scenario.

Table S10. Item diagnostics for prespecified indices with very low or negative Cronbach alpha.

Group	Domain	Item	n	Corrected item-total r	Alpha if deleted	Standardized alpha if deleted	Item wording
Patient	Accessibility	P17	273	0.068	0.268	0.242	How often have you been able to speak to someone immediately when you called the Fertility Department?
Patient	Accessibility	P19	259	0.084	0.239	0.233	Was it a problem for you to contact the staff (by phone or email, via WhatsApp) if you had any questions?
Patient	Accessibility	P101	273	0.108	0.221	0.210	How often have you had to wait more than 3 weeks to get an appointment with a doctor?
Patient	Accessibility	P103	273	0.172	0.157	0.200	How much time passed between your first hospital visit and when you received your treatment plan?
Patient	Accessibility	P105	226	0.159	0.163	0.206	How long on average did you have to wait 'unnecessarily' before being able to start a second treatment?
Patient	Privacy	P109	250	0.244	-0.030	0.089	Will this medical facility provide a female doctor for examination if you request it?
Patient	Privacy	P111	272	0.319	-0.144	0.015	To what extent do you feel your privacy life was respected?
Patient	Privacy	P113	213	0.106	0.151	0.193	Did your doctor or nurse ask your permission before allowing others to enter the room or procedure room?
Patient	Privacy	P115	215	-0.155	0.526	0.584	Has your doctor or nurse talked to others about your infertility problems?
Patient	Participation in Care	P54	272	-0.145	0.039	0.040	How often was your doctor open to your opinions and ideas about treatment?
Patient	Participation in Care	P55	273	-0.134	-0.088	-0.092	How often were you given the opportunity to ask questions to your doctor?
Patient	Participation in Care	P57	273	-0.029	-0.379	-0.439	Were you involved in decision making if you preferred?
Staff	Accessibility	S2	69	-0.008	0.255	0.184	-
Staff	Accessibility	S44	74	-0.032	0.210	0.188	-
Staff	Accessibility	S45	74	0.092	0.096	0.027	-
Staff	Accessibility	S46	68	0.233	-0.208	-0.131	-
Staff	Privacy	S48	27	0.060	0.210	0.166	-
Staff	Privacy	S49	68	0.122	0.114	0.177	-

Staff	Privacy	S50	70	0.276	-0.166	-0.019	-
Staff	Privacy	S51	67	-0.075	0.314	0.293	-
Staff	Staff Attitude and Communication	S14	74	0.333	0.245	0.321	-
Staff	Staff Attitude and Communication	S15	74	0.330	0.301	0.348	-
Staff	Staff Attitude and Communication	S16	74	0.217	0.307	0.396	-
Staff	Staff Attitude and Communication	S17	74	0.264	0.262	0.368	-
Staff	Staff Attitude and Communication	S18	74	0.074	0.393	0.502	-
Staff	Staff Attitude and Communication	S19	73	0.025	0.481	0.518	-
Staff	Staff Competence	S38	74	0.032	0.385	0.379	-
Staff	Staff Competence	S39	74	0.159	0.243	0.225	-
Staff	Staff Competence	S40	74	0.355	-0.122	-0.112	-
Staff	Staff Competence	S41	74	0.098	0.301	0.290	-
Staff	Continuity and Coordination	S31	63	-0.052	0.316	0.179	-
Staff	Continuity and Coordination	S32	74	-0.212	0.353	0.283	-
Staff	Continuity and Coordination	S33	64	0.204	0.095	0.078	-
Staff	Continuity and Coordination	S34	73	0.254	0.048	0.035	-
Staff	Continuity and Coordination	S35	74	-0.383	0.392	0.401	-
Staff	Continuity and Coordination	S36	74	0.458	-0.018	-0.124	-
Staff	Continuity and Coordination	S37	74	0.543	-0.196	-0.222	-
Staff	Continuity and Coordination	S42	74	-0.089	0.251	0.217	-
Staff	Participation in Care	S21	74	0.115	0.366	0.377	-
Staff	Participation in Care	S22	74	0.227	0.093	0.099	-
Staff	Participation in Care	S23	74	0.185	0.215	0.215	-

Footnote: Diagnostics are descriptive. Items were not removed post hoc to increase alpha.

Table S11. Pairwise inter-item correlations for prespecified indices with very low or negative Cronbach alpha.

Group	Domain	Item 1	Item 2	Pairwise n	Pearson r
Patient	Accessibility	score_p17	score_p19	259	0.205
Patient	Accessibility	score_p17	score_p101	273	0.025
Patient	Accessibility	score_p19	score_p101	259	0.160
Patient	Accessibility	score_p17	score_p103	273	-0.055
Patient	Accessibility	score_p19	score_p103	259	-0.033
Patient	Accessibility	score_p101	score_p103	273	0.063
Patient	Accessibility	score_p17	score_p105	226	0.033
Patient	Accessibility	score_p19	score_p105	216	-0.102
Patient	Accessibility	score_p101	score_p105	226	0.031
Patient	Accessibility	score_p103	score_p105	226	0.326
Patient	Privacy	score_p109	score_p111	249	0.263
Patient	Privacy	score_p109	score_p113	198	0.437
Patient	Privacy	score_p111	score_p113	212	0.257
Patient	Privacy	score_p109	score_p115	194	-0.150
Patient	Privacy	score_p111	score_p115	214	0.110
Patient	Privacy	score_p113	score_p115	166	-0.272
Patient	Participation in Care	score_p54	score_p55	272	-0.180
Patient	Participation in Care	score_p54	score_p57	272	-0.044
Patient	Participation in Care	score_p55	score_p57	273	0.020
Staff	Accessibility	score_s2	score_s44	69	-0.015
Staff	Accessibility	score_s2	score_s45	69	-0.147
Staff	Accessibility	score_s44	score_s45	74	0.041
Staff	Accessibility	score_s2	score_s46	63	0.117
Staff	Accessibility	score_s44	score_s46	68	-0.076
Staff	Accessibility	score_s45	score_s46	68	0.245
Staff	Privacy	score_s48	score_s49	22	0.243
Staff	Privacy	score_s48	score_s50	24	0.129
Staff	Privacy	score_s49	score_s50	65	-0.007
Staff	Privacy	score_s48	score_s51	21	-0.192
Staff	Privacy	score_s49	score_s51	63	-0.070
Staff	Privacy	score_s50	score_s51	65	0.264
Staff	Staff Attitude and Communication	score_s14	score_s15	74	0.317
Staff	Staff Attitude and Communication	score_s14	score_s16	74	0.324
Staff	Staff Attitude and Communication	score_s15	score_s16	74	0.209
Staff	Staff Attitude and Communication	score_s14	score_s17	74	0.326
Staff	Staff Attitude and Communication	score_s15	score_s17	74	0.242

Staff	Staff Attitude and Communication	score_s16	score_s17	74	0.332
Staff	Staff Attitude and Communication	score_s14	score_s18	74	0.085
Staff	Staff Attitude and Communication	score_s15	score_s18	74	0.059
Staff	Staff Attitude and Communication	score_s16	score_s18	74	-0.037
Staff	Staff Attitude and Communication	score_s17	score_s18	74	-0.086
Staff	Staff Attitude and Communication	score_s14	score_s19	73	-0.047
Staff	Staff Attitude and Communication	score_s15	score_s19	73	0.078
Staff	Staff Attitude and Communication	score_s16	score_s19	73	-0.117
Staff	Staff Attitude and Communication	score_s17	score_s19	73	0.013
Staff	Staff Attitude and Communication	score_s18	score_s19	73	0.171
Staff	Staff Competence	score_s38	score_s39	74	-0.069
Staff	Staff Competence	score_s38	score_s40	74	0.134
Staff	Staff Competence	score_s39	score_s40	74	0.294
Staff	Staff Competence	score_s38	score_s41	74	-0.058
Staff	Staff Competence	score_s39	score_s41	74	0.023
Staff	Staff Competence	score_s40	score_s41	74	0.189
Staff	Continuity and Coordination	score_s31	score_s32	63	-0.146
Staff	Continuity and Coordination	score_s31	score_s33	56	-0.057
Staff	Continuity and Coordination	score_s32	score_s33	64	-0.375
Staff	Continuity and Coordination	score_s31	score_s34	62	-0.157
Staff	Continuity and Coordination	score_s32	score_s34	73	-0.179
Staff	Continuity and Coordination	score_s33	score_s34	63	0.621
Staff	Continuity and Coordination	score_s31	score_s35	63	0.082
Staff	Continuity and Coordination	score_s32	score_s35	74	0.127
Staff	Continuity and Coordination	score_s33	score_s35	64	-0.369
Staff	Continuity and Coordination	score_s34	score_s35	73	-0.535
Staff	Continuity and Coordination	score_s31	score_s36	63	0.029
Staff	Continuity and Coordination	score_s32	score_s36	74	-0.041
Staff	Continuity and Coordination	score_s33	score_s36	64	0.498
Staff	Continuity and Coordination	score_s34	score_s36	73	0.471
Staff	Continuity and Coordination	score_s35	score_s36	74	-0.425
Staff	Continuity and Coordination	score_s31	score_s37	63	0.069
Staff	Continuity and Coordination	score_s32	score_s37	74	0.054
Staff	Continuity and Coordination	score_s33	score_s37	64	0.390
Staff	Continuity and Coordination	score_s34	score_s37	73	0.494
Staff	Continuity and Coordination	score_s35	score_s37	74	-0.233
Staff	Continuity and Coordination	score_s36	score_s37	74	0.482
Staff	Continuity and Coordination	score_s31	score_s42	63	0.188
Staff	Continuity and Coordination	score_s32	score_s42	74	0.080

Staff	Continuity and Coordination	score_s33	score_s42	64	-0.317
Staff	Continuity and Coordination	score_s34	score_s42	73	-0.183
Staff	Continuity and Coordination	score_s35	score_s42	74	0.164
Staff	Continuity and Coordination	score_s36	score_s42	74	-0.035
Staff	Continuity and Coordination	score_s37	score_s42	74	-0.054
Staff	Participation in Care	score_s21	score_s22	74	0.121
Staff	Participation in Care	score_s21	score_s23	74	0.052
Staff	Participation in Care	score_s22	score_s23	74	0.232

Table S12. Item-level scoring crosswalk, unexpected-code handling, and scoring reconciliation.

Panel A. Item-level scoring crosswalk.

Panel A1. Patient experience items.

Domain	Item	Source variable	Score type	Raw-to-analysis mapping	Documented nonanalytic codes	Unexpected n; value(s)
Accessibility	P101	p101_exp_raw	neg4	1->3; 2->2; 3->1; 4->0	None	0
Accessibility	P103	p103_exp_raw	wait_plan	1->0; 2->0.75; 3->1.5; 4->2.25; 5->3	None	0
Accessibility	P105	p105_exp_raw	wait_second	1->0; 2->1; 3->2; 4->3	5 (not applicable)	0
Accessibility	P17	p17_exp_raw	pos4	1->0; 2->1; 3->2; 4->3	None	0
Accessibility	P19	p19_exp_raw	contact_problem	1->0; 2->1.5; 3->3	4 (not applicable)	0
Continuity and Coordination	P75	p75_exp_raw	binary_pos	1->0; 2->3	None	0
Continuity and Coordination	P77	p77_exp_raw	num_doctors	1->3; 2->1.5; 3->0	None	0
Continuity and Coordination	P79	p79_exp_raw	lead_doctor	1->0; 2->1.5; 3->3	None	0
Continuity and Coordination	P81	p81_exp_raw	pos4	1->0; 2->1; 3->2; 4->3	None	0
Continuity and Coordination	P83	p83_exp_raw	neg4	1->3; 2->2; 3->1; 4->0	None	0
Continuity and Coordination	P85	p85_exp_raw	neg4	1->3; 2->2; 3->1; 4->0	None	0
Continuity and Coordination	P87	p87_exp_raw	neg4	1->3; 2->2; 3->1; 4->0	None	0
Continuity and Coordination	P97	p97_exp_raw	pos4	1->0; 2->1; 3->2; 4->3	None	0
Emotional and Psychological Support	P61	p61_exp_raw	record_access	1->0; 2->1.5; 3->3	4 (do not know)	0
Emotional and Psychological Support	P63	p63_exp_raw	pos4	1->0; 2->1; 3->2; 4->3	None	0
Emotional and Psychological Support	P65	p65_exp_raw	pos4	1->0; 2->1; 3->2; 4->3	None	0
Emotional and Psychological Support	P67	p67_exp_raw	pos4_na5	1->0; 2->1; 3->2; 4->3	5 (not applicable)	0
Emotional and Psychological Support	P69	p69_exp_raw	partner_involvement	1->0; 2->1; 3->2; 4->3	5 (not applicable)	0

Emotional and Psychological Support	P71	p71_exp_raw	pos4_na5	1->0; 2->1; 3->2; 4->3	5 (not applicable)	0
Emotional and Psychological Support	P73	p73_exp_raw	emotional_attention	1->0; 2->1; 3->2; 4->3	5 (not applicable)	0
Information and Education	P21	p21_exp_raw	binary_pos	1->0; 2->3	None	6; 3;4
Information and Education	P23	p23_exp_raw	written_info	1->0; 2->1.5; 3->3; 4->1.5; 5->0	None	0
Information and Education	P25	p25_exp_raw	pos4	1->0; 2->1; 3->2; 4->3	None	0
Information and Education	P26	p26_exp_raw	pos4	1->0; 2->1; 3->2; 4->3	None	0
Information and Education	P28	p28_exp_raw	pos4	1->0; 2->1; 3->2; 4->3	None	0
Information and Education	P30	p30_exp_raw	outline_yes_na	1->0; 2->3	3 (not applicable)	0
Information and Education	P32	p32_exp_raw	side_effect_info	1->0; 2->1.5; 3->3	4 (not applicable)	0
Information and Education	P34	p34_exp_raw	pos4_na5	1->0; 2->1; 3->2; 4->3	5 (not applicable)	1; 0
Information and Education	P36	p36_exp_raw	three_pos	1->0; 2->1.5; 3->3	None	1; 4
Information and Education	P38	p38_exp_raw	multi_select_exclude	Not scored	All observed codes	0
Minimizing Cost	P107	p107_exp_raw	cost3	1->0; 2->1.5; 3->3	None	0
Participation in Care	P54	p54_exp_raw	pos4	1->0; 2->1; 3->2; 4->3	None	1; 0
Participation in Care	P55	p55_exp_raw	pos4	1->0; 2->1; 3->2; 4->3	None	0
Participation in Care	P57	p57_exp_raw	pos4	1->0; 2->1; 3->2; 4->3	None	0
Physical Comfort proxy	P99	p99_exp_raw	waiting_room	1->0; 2->1; 3->2; 4->3	5 (not applicable)	0
Privacy	P109	p109_exp_raw	female_doctor	2->0; 3->1; 4->2; 5->3	1 (not applicable/did not ask)	0
Privacy	P111	p111_exp_raw	pos4	1->0; 2->1; 3->2; 4->3	None	1; 45
Privacy	P113	p113_exp_raw	permission_shift5	2->0; 3->1; 4->2; 5->3	1 (not applicable)	0
Privacy	P115	p115_exp_raw	confidentiality	1->0; 2->1; 3->2; 4->3	5 (not applicable)	1; 21
Staff Attitude and Communication	P42	p42_exp_raw	pos4	1->0; 2->1; 3->2; 4->3	None	1; 0
Staff Attitude and Communication	P44	p44_exp_raw	three_pos	1->0; 2->1.5; 3->3	None	9; 4

Staff Attitude and Communication	P46	p46_exp_raw	pos4	1->0; 2->1; 3->2; 4->3	None	0
Staff Attitude and Communication	P48	p48_exp_raw	pos4	1->0; 2->1; 3->2; 4->3	None	1; 0
Staff Attitude and Communication	P50	p50_exp_raw	pos4	1->0; 2->1; 3->2; 4->3	None	2; 0
Staff Attitude and Communication	P52	p52_exp_raw	neg4	1->3; 2->2; 3->1; 4->0	None	1; 0
Staff Attitude and Communication	P53	p53_exp_raw	error_discussion	1->0; 2->3	3 (nothing went wrong)	13; 4
Staff Competence	P89	p89_exp_raw	neg4	1->3; 2->2; 3->1; 4->0	None	0
Staff Competence	P91	p91_exp_raw	pos4	1->0; 2->1; 3->2; 4->3	None	0
Staff Competence	P93	p93_exp_raw	pos4	1->0; 2->1; 3->2; 4->3	None	0
Staff Competence	P95	p95_exp_raw	neg4	1->3; 2->2; 3->1; 4->0	None	0

Panel A2. Patient importance items.

Domain	Item	Source variable	Score type	Raw-to-analysis mapping	Documented nonanalytic codes	Unexpected n; value(s)
Accessibility	P102	p102_imp_raw	importance_reverse_4	1->3; 2->2; 3->1; 4->0	Any code outside 1-4	2; 0
Accessibility	P104	p104_imp_raw	importance_reverse_4	1->3; 2->2; 3->1; 4->0	Any code outside 1-4	5; 0;5
Accessibility	P106	p106_imp_raw	importance_reverse_4	1->3; 2->2; 3->1; 4->0	Any code outside 1-4	1; 0
Accessibility	P18	p18_imp_raw	importance_reverse_4	1->3; 2->2; 3->1; 4->0	Any code outside 1-4	0
Accessibility	P20	p20_imp_raw	importance_reverse_4	1->3; 2->2; 3->1; 4->0	Any code outside 1-4	0
Continuity and Coordination	P76	p76_imp_raw	importance_reverse_4	1->3; 2->2; 3->1; 4->0	Any code outside 1-4	0
Continuity and Coordination	P78	p78_imp_raw	importance_reverse_4	1->3; 2->2; 3->1; 4->0	Any code outside 1-4	2; 0
Continuity and Coordination	P80	p80_imp_raw	importance_reverse_4	1->3; 2->2; 3->1; 4->0	Any code outside 1-4	0
Continuity and Coordination	P82	p82_imp_raw	importance_reverse_4	1->3; 2->2; 3->1; 4->0	Any code outside 1-4	0
Continuity and Coordination	P84	p84_imp_raw	importance_reverse_4	1->3; 2->2; 3->1; 4->0	Any code outside 1-4	2; 0

Continuity and Coordination	P86	p86_imp_raw	importance_reverse_4	1->3; 2->2; 3->1; 4->0	Any code outside 1-4	2; 0
Continuity and Coordination	P88	p88_imp_raw	importance_reverse_4	1->3; 2->2; 3->1; 4->0	Any code outside 1-4	0
Continuity and Coordination	P98	p98_imp_raw	importance_reverse_4	1->3; 2->2; 3->1; 4->0	Any code outside 1-4	0
Emotional and Psychological Support	P62	p62_imp_raw	importance_reverse_4	1->3; 2->2; 3->1; 4->0	Any code outside 1-4	0
Emotional and Psychological Support	P64	p64_imp_raw	importance_reverse_4	1->3; 2->2; 3->1; 4->0	Any code outside 1-4	0
Emotional and Psychological Support	P66	p66_imp_raw	importance_reverse_4	1->3; 2->2; 3->1; 4->0	Any code outside 1-4	1; 0
Emotional and Psychological Support	P68	p68_imp_raw	importance_reverse_4	1->3; 2->2; 3->1; 4->0	Any code outside 1-4	0
Emotional and Psychological Support	P70	p70_imp_raw	importance_reverse_4	1->3; 2->2; 3->1; 4->0	Any code outside 1-4	0
Emotional and Psychological Support	P72	p72_imp_raw	importance_reverse_4	1->3; 2->2; 3->1; 4->0	Any code outside 1-4	2; 5
Emotional and Psychological Support	P74	p74_imp_raw	importance_reverse_4	1->3; 2->2; 3->1; 4->0	Any code outside 1-4	0
Information and Education	P22	p22_imp_raw	importance_reverse_4	1->3; 2->2; 3->1; 4->0	Any code outside 1-4	1; 0
Information and Education	P24	p24_imp_raw	importance_reverse_4	1->3; 2->2; 3->1; 4->0	Any code outside 1-4	1; 0
Information and Education	P27	p27_imp_raw	importance_reverse_4	1->3; 2->2; 3->1; 4->0	Any code outside 1-4	0
Information and Education	P29	p29_imp_raw	importance_reverse_4	1->3; 2->2; 3->1; 4->0	Any code outside 1-4	1; 0
Information and Education	P31	p31_imp_raw	importance_reverse_4	1->3; 2->2; 3->1; 4->0	Any code outside 1-4	2; 0
Information and Education	P33	p33_imp_raw	importance_reverse_4	1->3; 2->2; 3->1; 4->0	Any code outside 1-4	1; 0
Information and Education	P35	p35_imp_raw	importance_reverse_4	1->3; 2->2; 3->1; 4->0	Any code outside 1-4	0
Information and Education	P37	p37_imp_raw	importance_reverse_4	1->3; 2->2; 3->1; 4->0	Any code outside 1-4	0
Information and Education	P39	p39_imp_raw	importance_reverse_4	1->3; 2->2; 3->1; 4->0	Any code outside 1-4	206; 5;6;7;78

Minimizing Cost	P108	p108_imp_raw	importance_reverse_4	1->3; 2->2; 3->1; 4->0	Any code outside 1-4	2; 5
Participation in Care	P56	p56_imp_raw	importance_reverse_4	1->3; 2->2; 3->1; 4->0	Any code outside 1-4	1; 0
Participation in Care	P58	p58_imp_raw	importance_reverse_4	1->3; 2->2; 3->1; 4->0	Any code outside 1-4	1; 0
Physical Comfort proxy	P100	p100_imp_raw	importance_reverse_4	1->3; 2->2; 3->1; 4->0	Any code outside 1-4	0
Privacy	P110	p110_imp_raw	importance_reverse_4	1->3; 2->2; 3->1; 4->0	Any code outside 1-4	0
Privacy	P112	p112_imp_raw	importance_reverse_4	1->3; 2->2; 3->1; 4->0	Any code outside 1-4	1; 5
Privacy	P114	p114_imp_raw	importance_reverse_4	1->3; 2->2; 3->1; 4->0	Any code outside 1-4	0
Privacy	P116	p116_imp_raw	importance_reverse_4	1->3; 2->2; 3->1; 4->0	Any code outside 1-4	0
Staff Attitude and Communication	P43	p43_imp_raw	importance_reverse_4	1->3; 2->2; 3->1; 4->0	Any code outside 1-4	0
Staff Attitude and Communication	P45	p45_imp_raw	importance_reverse_4	1->3; 2->2; 3->1; 4->0	Any code outside 1-4	0
Staff Attitude and Communication	P47	p47_imp_raw	importance_reverse_4	1->3; 2->2; 3->1; 4->0	Any code outside 1-4	4; 10
Staff Attitude and Communication	P49	p49_imp_raw	importance_reverse_4	1->3; 2->2; 3->1; 4->0	Any code outside 1-4	1; 0
Staff Attitude and Communication	P51	p51_imp_raw	importance_reverse_4	1->3; 2->2; 3->1; 4->0	Any code outside 1-4	0
Staff Competence	P90	p90_imp_raw	importance_reverse_4	1->3; 2->2; 3->1; 4->0	Any code outside 1-4	0
Staff Competence	P92	p92_imp_raw	importance_reverse_4	1->3; 2->2; 3->1; 4->0	Any code outside 1-4	0
Staff Competence	P94	p94_imp_raw	importance_reverse_4	1->3; 2->2; 3->1; 4->0	Any code outside 1-4	1; 0
Staff Competence	P96	p96_imp_raw	importance_reverse_4	1->3; 2->2; 3->1; 4->0	Any code outside 1-4	0

Panel A3. Staff experience items.

Domain	Item	Source variable	Score type	Raw-to-analysis mapping	Documented nonanalytic codes	Unexpected n; value(s)

Accessibility	S1	s01_exp_raw	pos4	1->0; 2->1; 3->2; 4->3	None	0
Accessibility	S2	s02_exp_raw	contact_problem	1->0; 2->1.5; 3->3	4 (not applicable)	0
Accessibility	S44	s44_exp_raw	neg4	1->3; 2->2; 3->1; 4->0	None	0
Accessibility	S45	s45_exp_raw	wait_plan	1->0; 2->0.75; 3->1.5; 4->2.25; 5->3	None	0
Accessibility	S46	s46_exp_raw	wait_second	1->0; 2->1; 3->2; 4->3	5 (not applicable)	0
Continuity and Coordination	S31	s31_exp_raw	binary_pos	1->0; 2->3	None	11; 3;4
Continuity and Coordination	S32	s32_exp_raw	num_doctors	1->3; 2->1.5; 3->0	None	0
Continuity and Coordination	S33	s33_exp_raw	lead_doctor	1->0; 2->1.5; 3->3	None	10; 4
Continuity and Coordination	S34	s34_exp_raw	pos4	1->0; 2->1; 3->2; 4->3	None	1; 0
Continuity and Coordination	S35	s35_exp_raw	neg4	1->3; 2->2; 3->1; 4->0	None	0
Continuity and Coordination	S36	s36_exp_raw	neg4	1->3; 2->2; 3->1; 4->0	None	0
Continuity and Coordination	S37	s37_exp_raw	neg4	1->3; 2->2; 3->1; 4->0	None	0
Continuity and Coordination	S42	s42_exp_raw	pos4	1->0; 2->1; 3->2; 4->3	None	0
Emotional and Psychological Support	S24	s24_exp_raw	record_access	1->0; 2->1.5; 3->3	4 (do not know)	0
Emotional and Psychological Support	S25	s25_exp_raw	pos4	1->0; 2->1; 3->2; 4->3	None	2; 5
Emotional and Psychological Support	S26	s26_exp_raw	pos4	1->0; 2->1; 3->2; 4->3	None	0
Emotional and Psychological Support	S27	s27_exp_raw	pos4_na5	1->0; 2->1; 3->2; 4->3	5 (not applicable)	0
Emotional and Psychological Support	S28	s28_exp_raw	partner_involvement	1->0; 2->1; 3->2; 4->3	5 (not applicable)	0
Emotional and Psychological Support	S29	s29_exp_raw	pos4_na5	1->0; 2->1; 3->2; 4->3	5 (not applicable)	0

Emotional and Psychological Support	S30	s30_exp_raw	emotional_attention	1->0; 2->1; 3->2; 4->3	5 (not applicable)	0
Information and Education	S10	s10_exp_raw	pos4_na5	1->0; 2->1; 3->2; 4->3	5 (not applicable)	0
Information and Education	S11	s11_exp_raw	three_pos	1->0; 2->1.5; 3->3	None	0
Information and Education	S12	s12_exp_raw	multi_select_exclude	Not scored	All observed codes	0
Information and Education	S3	s03_exp_raw	binary_pos	1->0; 2->3	None	0
Information and Education	S4	s04_exp_raw	written_info	1->0; 2->1.5; 3->3; 4->1.5; 5->0	None	0
Information and Education	S5	s05_exp_raw	pos4	1->0; 2->1; 3->2; 4->3	None	0
Information and Education	S6	s06_exp_raw	pos4	1->0; 2->1; 3->2; 4->3	None	0
Information and Education	S7	s07_exp_raw	pos4	1->0; 2->1; 3->2; 4->3	None	0
Information and Education	S8	s08_exp_raw	outline_yes_na	1->0; 2->3	3 (not applicable)	0
Information and Education	S9	s09_exp_raw	side_effect_info	1->0; 2->1.5; 3->3	4 (not applicable)	0
Minimizing Cost	S47	s47_exp_raw	cost3	1->0; 2->1.5; 3->3	None	0
Participation in Care	S21	s21_exp_raw	pos4	1->0; 2->1; 3->2; 4->3	None	0
Participation in Care	S22	s22_exp_raw	pos4	1->0; 2->1; 3->2; 4->3	None	0
Participation in Care	S23	s23_exp_raw	pos4	1->0; 2->1; 3->2; 4->3	None	0
Physical Comfort proxy	S43	s43_exp_raw	waiting_room	1->0; 2->1; 3->2; 4->3	5 (not applicable)	0
Privacy	S48	s48_exp_raw	female_doctor	2->0; 3->1; 4->2; 5->3	1 (not applicable/did not ask)	0
Privacy	S49	s49_exp_raw	pos4	1->0; 2->1; 3->2; 4->3	None	6; 5
Privacy	S50	s50_exp_raw	permission_shift5	2->0; 3->1; 4->2; 5->3	1 (not applicable)	0

Privacy	S51	s51_exp_raw	confidentiality	1->0; 2->1; 3->2; 4->3	5 (not applicable)	0
Staff Attitude and Communication	S14	s14_exp_raw	pos4	1->0; 2->1; 3->2; 4->3	None	0
Staff Attitude and Communication	S15	s15_exp_raw	three_pos	1->0; 2->1.5; 3->3	None	0
Staff Attitude and Communication	S16	s16_exp_doctor_raw	pos4	1->0; 2->1; 3->2; 4->3	None	0
Staff Attitude and Communication	S16	s16_exp_embryologist_raw	pos4	1->0; 2->1; 3->2; 4->3	None	0
Staff Attitude and Communication	S16	s16_exp_lab_raw	pos4	1->0; 2->1; 3->2; 4->3	None	0
Staff Attitude and Communication	S16	s16_exp_nurse_raw	pos4	1->0; 2->1; 3->2; 4->3	None	0
Staff Attitude and Communication	S16	s16_exp_reception_raw	pos4	1->0; 2->1; 3->2; 4->3	None	0
Staff Attitude and Communication	S17	s17_exp_doctor_raw	pos4	1->0; 2->1; 3->2; 4->3	None	0
Staff Attitude and Communication	S17	s17_exp_embryologist_raw	pos4	1->0; 2->1; 3->2; 4->3	None	0
Staff Attitude and Communication	S17	s17_exp_lab_raw	pos4	1->0; 2->1; 3->2; 4->3	None	0
Staff Attitude and Communication	S17	s17_exp_nurse_raw	pos4	1->0; 2->1; 3->2; 4->3	None	0
Staff Attitude and Communication	S17	s17_exp_reception_raw	pos4	1->0; 2->1; 3->2; 4->3	None	0
Staff Attitude and Communication	S18	s18_exp_doctor_raw	pos4	1->0; 2->1; 3->2; 4->3	None	0
Staff Attitude and Communication	S18	s18_exp_embryologist_raw	pos4	1->0; 2->1; 3->2; 4->3	None	0
Staff Attitude and Communication	S18	s18_exp_lab_raw	pos4	1->0; 2->1; 3->2; 4->3	None	0
Staff Attitude and Communication	S18	s18_exp_nurse_raw	pos4	1->0; 2->1; 3->2; 4->3	None	0
Staff Attitude and Communication	S18	s18_exp_reception_raw	pos4	1->0; 2->1; 3->2; 4->3	None	0
Staff Attitude and Communication	S19	s19_exp_raw	neg4	1->3; 2->2; 3->1; 4->0	None	0

Staff Attitude and Communication	S20	s20_exp_raw	error_discussion	1->0; 2->3	3 (nothing went wrong)	0
Staff Competence	S38	s38_exp_raw	neg4	1->3; 2->2; 3->1; 4->0	None	0
Staff Competence	S39	s39_exp_raw	pos4	1->0; 2->1; 3->2; 4->3	None	0
Staff Competence	S40	s40_exp_raw	pos4	1->0; 2->1; 3->2; 4->3	None	0
Staff Competence	S41	s41_exp_raw	neg4	1->3; 2->2; 3->1; 4->0	None	0

Panel A4. Patient and staff global ratings.

Domain	Item	Source variable	Score type	Raw-to-analysis mapping	Documented nonanalytic codes	Unexpected n; value(s)
Global Care Rating	Global rating	global_rating_raw	global_0_to_10	0-10 retained	Any code outside 0-10	0
Global Care Rating	Global rating	staff_global_rating_raw	global_0_to_10	0-10 retained	Any code outside 0-10	50; 11

Panel B. Unexpected raw codes and analytical treatment.

Group	Item	Score type	Unexpected n	Unexpected values	Analysis component	Meaning	Analytical treatment
Patient	P111	pos4	1	45	Patient experience	Undocumented	Set missing for this item
Staff	S49	pos4	6	5	Staff experience	Undocumented	Set missing for this item
Patient	P115	confidentiality	1	21	Patient experience	Undocumented	Set missing for this item
Patient	P42	pos4	1	0	Patient experience	Undocumented	Set missing for this item
Patient	P44	three_pos	9	4	Patient experience	Undocumented	Set missing for this item
Patient	P48	pos4	1	0	Patient experience	Undocumented	Set missing for this item
Patient	P50	pos4	2	0	Patient experience	Undocumented	Set missing for this item
Patient	P52	neg4	1	0	Patient experience	Undocumented	Set missing for this item

Patient	P53	error_discussion	13	4	Patient experience	Undocumented	Set missing for this item
Patient	P21	binary_pos	6	3;4	Patient experience	Undocumented	Set missing for this item
Patient	P34	pos4_na5	1	0	Patient experience	Undocumented	Set missing for this item
Patient	P36	three_pos	1	4	Patient experience	Undocumented	Set missing for this item
Staff	S25	pos4	2	5	Staff experience	Undocumented	Set missing for this item
Staff	S31	binary_pos	11	3;4	Staff experience	Undocumented	Set missing for this item
Staff	S33	lead_doctor	10	4	Staff experience	Undocumented	Set missing for this item
Staff	S34	pos4	1	0	Staff experience	Undocumented	Set missing for this item
Patient	P54	pos4	1	0	Patient experience	Undocumented	Set missing for this item
Patient	P22	importance_reverse_4	1	0	Patient importance	Undocumented	Set missing for this item
Patient	P24	importance_reverse_4	1	0	Patient importance	Undocumented	Set missing for this item
Patient	P29	importance_reverse_4	1	0	Patient importance	Undocumented	Set missing for this item
Patient	P31	importance_reverse_4	2	0	Patient importance	Undocumented	Set missing for this item
Patient	P33	importance_reverse_4	1	0	Patient importance	Undocumented	Set missing for this item
Patient	P39	importance_reverse_4	206	5;6;7;78	Patient importance	Undocumented	Set missing for this item
Patient	P47	importance_reverse_4	4	10	Patient importance	Undocumented	Set missing for this item
Patient	P49	importance_reverse_4	1	0	Patient importance	Undocumented	Set missing for this item
Patient	P56	importance_reverse_4	1	0	Patient importance	Undocumented	Set missing for this item
Patient	P58	importance_reverse_4	1	0	Patient importance	Undocumented	Set missing for this item

Patient	P66	importance_reverse_4	1	0	Patient importance	Undocumented	Set missing for this item
Patient	P72	importance_reverse_4	2	5	Patient importance	Undocumented	Set missing for this item
Patient	P78	importance_reverse_4	2	0	Patient importance	Undocumented	Set missing for this item
Patient	P84	importance_reverse_4	2	0	Patient importance	Undocumented	Set missing for this item
Patient	P86	importance_reverse_4	2	0	Patient importance	Undocumented	Set missing for this item
Patient	P94	importance_reverse_4	1	0	Patient importance	Undocumented	Set missing for this item
Patient	P102	importance_reverse_4	2	0	Patient importance	Undocumented	Set missing for this item
Patient	P104	importance_reverse_4	5	0;5	Patient importance	Undocumented	Set missing for this item
Patient	P106	importance_reverse_4	1	0	Patient importance	Undocumented	Set missing for this item
Patient	P108	importance_reverse_4	2	5	Patient importance	Undocumented	Set missing for this item
Patient	P112	importance_reverse_4	1	5	Patient importance	Undocumented	Set missing for this item
Staff	Global rating	global_0_to_10	50	11	Staff global rating	Undocumented	Code 11 set missing; staff global rating excluded

Panel C. Experience items with <95% exact agreement.

Domain	Patient item	Score type	Provided column	Comparable n	Exact agreement n	Agreement %
Accessibility	Q17	pos4	p17_exp_provided_score	273	69	25.3
Accessibility	Q101	neg4	p101_exp_provided_score	273	0	0.0
Accessibility	Q103	wait_plan	p103_exp_provided_score	272	77	28.3
Minimizing Cost	Q107	cost3	p107_exp_provided_score	273	122	44.7
Privacy	Q109	female_doctor	p109_exp_provided_score	250	0	0.0
Privacy	Q113	permission_shift5	p113_exp_provided_score	213	0	0.0
Privacy	Q115	confidentiality	p115_exp_provided_score	214	1	0.5
Staff Attitude and Communication	Q42	pos4	p42_exp_provided_score	272	171	62.9
Staff Attitude and Communication	Q44	three_pos	p44_exp_provided_score	264	147	55.7

Staff Attitude and Communication	Q46	pos4	p46_exp_provided_score	273	228	83.5
Staff Attitude and Communication	Q48	pos4	p48_exp_provided_score	272	255	93.8
Staff Attitude and Communication	Q50	pos4	p50_exp_provided_score	271	244	90.0
Staff Attitude and Communication	Q53	error_discussion	p53_exp_provided_score	246	217	88.2
Information and Education	Q21	binary_pos	p21_exp_provided_score	267	78	29.2
Information and Education	Q23	written_info	p23_exp_provided_score	273	240	87.9
Information and Education	Q26	pos4	p26_exp_provided_score	273	217	79.5
Information and Education	Q30	outline_yes_na	p30_exp_provided_score	242	148	61.2
Information and Education	Q32	side_effect_info	p32_exp_provided_score	272	149	54.8
Information and Education	Q36	three_pos	p36_exp_provided_score	272	174	64.0
Emotional and Psychological Support	Q61	record_access	p61_exp_provided_score	122	28	23.0
Continuity and Coordination	Q75	binary_pos	p75_exp_provided_score	273	72	26.4
Continuity and Coordination	Q77	num_doctors	p77_exp_provided_score	273	0	0.0
Continuity and Coordination	Q79	lead_doctor	p79_exp_provided_score	273	243	89.0
Continuity and Coordination	Q83	neg4	p83_exp_provided_score	273	0	0.0
Continuity and Coordination	Q85	neg4	p85_exp_provided_score	273	42	15.4
Participation in Care	Q54	pos4	p54_exp_provided_score	272	1	0.4

Panel D. Importance items with <95% exact agreement.

Domain	Importance item	Provided column	Comparable n	Exact agreement n	Agreement %
Minimizing Cost	Q108	p108_imp_provided_score	271	15	5.5
Staff Attitude and Communication	Q49	p49_imp_provided_score	272	219	80.5
Information and Education	Q37	p37_imp_provided_score	273	0	0.0
Information and Education	Q39	p39_imp_provided_score	66	0	0.0

Footnote: Panel A documents the mapping implemented in Supplementary Code S1 for every source variable included in the scoring audit. Panel B lists each variable with at least one value outside its implemented scoring set. The meaning of every listed unexpected value was undocumented in the available codebook and archived materials. Unexpected item values were set to missing, and code 11 for the staff global rating was set to missing before that outcome was excluded. The audit identified 308 unexpected item responses, comprising 278 patient and 30 staff responses, plus 50 staff global ratings coded 11. The patient count included 240 importance responses, of which 206 occurred in P39; because its corresponding experience item P38 was not scored, P39 was not included in Table S4. Disagreement with legacy conversion columns does not establish which mapping is correct. Definitive reconciliation requires the administered item-level codebook.

Supplementary Figures

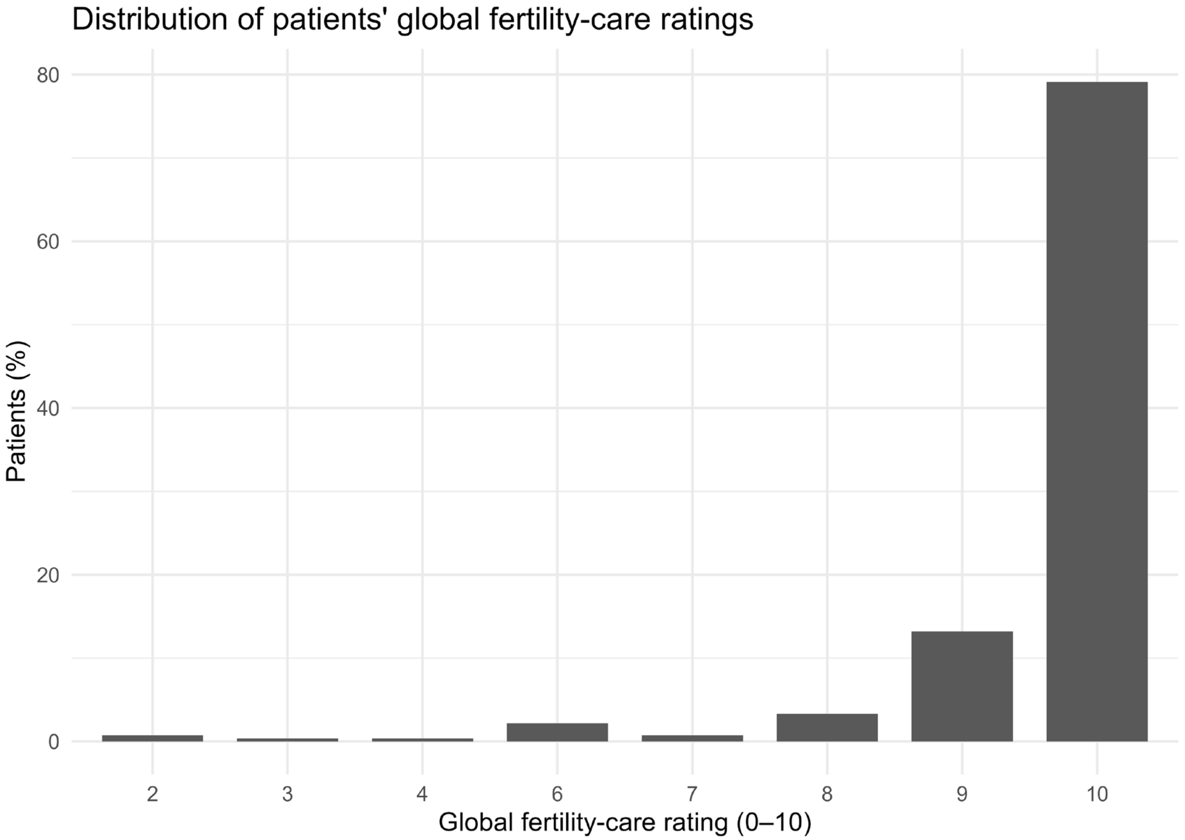

Figure S1. Distribution of the patient global fertility-care rating. The maximum rating of 10 accounted for 79.1% of patient records.

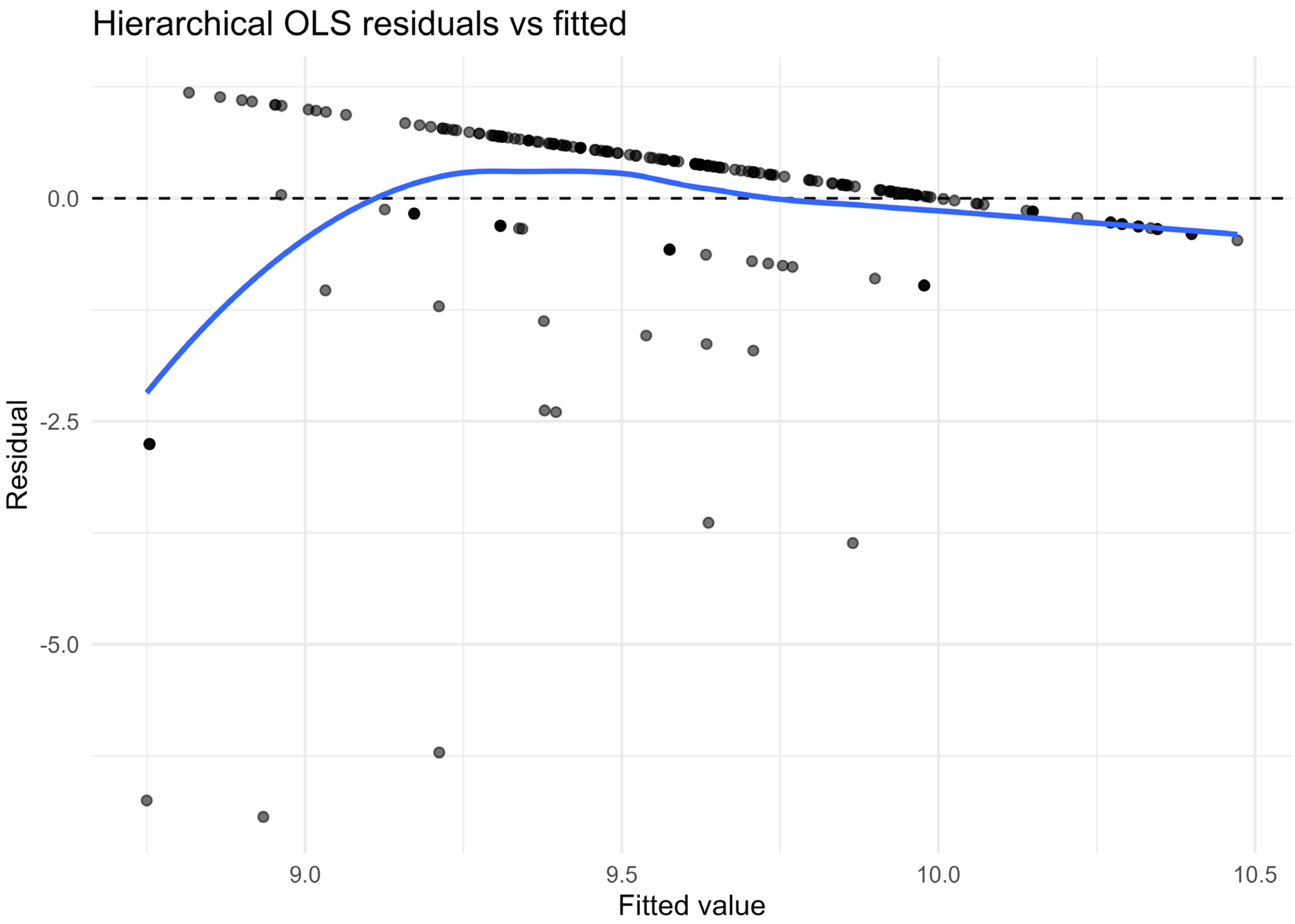

Figure S2. Exploratory linear-model residual diagnostics. The bounded ceiling-skewed outcome limits the interpretation of ordinary least-squares residual diagnostics and coefficients.

Supplementary Code S1. R code for data processing, scoring, statistical analyses, sensitivity analyses, and generation of tables and figures
